# Advancements in intratumoral therapies for liver tumors

**DOI:** 10.3389/fonc.2026.1726128

**Published:** 2026-02-04

**Authors:** Steven S. Raman, Neal R. Cutler, John J. Sramek, J. Randolph Hecht, Richard S. Finn, Sidharth R. Anand, Jason Chiang, David S. Lu

**Affiliations:** 1 Radiology, Department of Radiological Sciences Director, University of California, Los Angeles (UCLA) Uterine Fibroid Program, University of California, Los Angeles (UCLA) Healthcare, Los Angeles, CA, United States; 2 Abdominal Imaging Fellowship Program, University of California, Los Angeles (UCLA) Healthcare, Los Angeles, CA, United States; 3 Concierge Clinical Trials, Beverly Hills, CA, United States; 4 Clinical Research, Concierge Clinical Trials, Beverly Hills, CA, United States; 5 Clinical Medicine, David Geffen School of Medicine at UCLA, Los Angeles, CA, United States; 6 University of California, Los Angeles Gastrointestinal Oncology Program, University of California, Los Angeles (UCLA) Healthcare, Los Angeles, CA, United States; 7 Department of Medicine, Hematology/Oncology, University of California, Los Angeles (UCLA) Healthcare, Los Angeles, CA, United States; 8 Department of Surgery, Dumont-University of California, Los Angeles Liver Cancer Center, University of California, Los Angeles (UCLA) Healthcare, Los Angeles, CA, United States; 9 Signal Transduction and Therapeutics, University of California, Los Angeles (UCLA) Healthcare, Los Angeles, CA, United States; 10 Department of Medicine - Hematology/Oncology, University of California, Los Angeles (UCLA) Healthcare, Los Angeles, CA, United States; 11 Abdominal Imaging and Interventional Radiology, University of California, Los Angeles (UCLA) Healthcare, Los Angeles, CA, United States; 12 Radiology and Surgery, Abdominal Imaging and Intervention Director, Computed Tomography, University of California, Los Angeles (UCLA) Healthcare, Los Angeles, CA, United States; 13 Interventional Oncology, University of California, Los Angeles (UCLA) Healthcare, Los Angeles, CA, United States

**Keywords:** hepatocellular carcinoma, immunotherapy, intratumoral therapy, liver metastasis, locoregional treatment, oncolytic virotherapy, tumor microenvironment

## Abstract

Liver tumors, including hepatocellular carcinoma (HCC), intrahepatic cholangiocarcinoma (iCCA), and liver-dominant metastases, remain associated with high mortality despite advances in systemic therapy. Intratumoral therapies have emerged as a promising strategy to achieve high local drug concentrations, modulate the tumor microenvironment, and enhance systemic anti-tumor immunity while limiting systemic toxicity. Although intratumoral approaches have demonstrated clinical success in melanoma, their translation to liver malignancies presents unique biological, immunologic, and technical challenges. This review synthesizes clinical evidence from the past decade evaluating intratumoral therapies for primary and metastatic liver tumors, including oncolytic viruses, cell-based immunotherapies, *in situ* immunomodulators, intratumoral chemotherapy, and combination strategies with locoregional or systemic treatments. Across early- and late-phase trials, intratumoral therapies have produced heterogeneous outcomes, ranging from tumor necrosis and disease stabilization to occasional systemic (abscopal) responses, while several large studies have failed to demonstrate survival benefit. These mixed results reflect the liver’s highly tolerogenic immune microenvironment, characterized by abundant myeloid-derived suppressor cells, regulatory T cells, and abnormal vasculature that limit immune activation and drug distribution. We highlight key determinants of efficacy, including tumor biology, delivery technique, dosing strategy, and rational therapeutic combinations. Technical considerations such as image-guided injection, intratumoral pressure, and standardization of administration are reviewed, alongside emerging biomarkers, including immune, molecular, and imaging-based markers, that may enable improved patient selection. Overall, current evidence suggests that intratumoral therapies alone are rarely sufficient for liver tumors but may provide meaningful benefit when integrated into multimodal regimens. Future progress will depend on optimized combination strategies, standardized delivery approaches, and validated biomarkers to support personalized application in liver cancer.

## Introduction

### Epidemiology and outcomes of liver tumors

Liver tumors constitute a major global health burden. Primary liver cancer (hepatocellular carcinoma, HCC) is the sixth most common malignancy worldwide and the third leading cause of cancer-related death. Unfortunately, HCC is often detected late – only about 30% of patients are diagnosed at an early stage amenable to curative treatment by resection or transplantation (1). Consequently, overall survival remains poor. The five-year survival rate for liver cancer reflects its status as one of the most lethal tumors (2). In the past two decades, with improved screening and early detection amenable to curative treatments, as well as availability of new curative treatments, the survival for all stages has improved to the range of 30-40%. However, survival for late-stage metastatic HCC is still dismal. Although targeted therapies and immunotherapies have also improved 5-year survival of patients with advanced HCC, it is still low at around 20%. Survival of intrahepatic cholangiocarcinoma (iCCA) remains low with median overall survival less than 10% (3).

The liver is a common site for cancer spread and liver metastases. For example, the liver is the most common visceral site of metastasis from colorectal cancer (CRC), and ~50% of CRC patients will develop liver metastases during the disease (4). Prognosis for metastatic liver tumors is similarly guarded. Modern combination chemotherapy and targeted agents have improved outcomes in metastatic CRC, but five-year survival still averages only around 15–20% (5). Notably, a subset of patients can achieve long-term survival if aggressive treatment is feasible; surgical metastasectomy can cure roughly 20% of patients and over half of those undergoing resection and/or ablation (6) survive beyond five years (4). These epidemiologic and outcome data underscore the high mortality associated with both primary and secondary liver tumors (2, 5), and they frame the urgent need for more effective therapeutic strategies.

### Intratumoral therapy in the broader oncology context: successes and challenges

There are several theoretical advantages driving interest in intratumoral approaches. First, local administration can achieve drug concentrations in the tumor that would be toxic if given systemically. Potent therapeutics that are limited by systemic toxicity might be safely delivered into the tumor to maximize efficacy *in-situ* (7). Second, intratumoral injection can disrupt the immunosuppressive tumor microenvironment. By injecting immune-stimulating agents directly into the lesion, one can provoke a robust local immune attack, effectively turning the tumor into an *in-situ* vaccine that generates systemic T-cell responses against tumor antigens (7). The latter concept led to the development of talimogene laherparepvec (T-VEC, an HSV-1 based oncolytic virus), which became the first FDA approved intratumoral therapy for peripheral melanoma (8). Third, improved local tumor control from intratumoral therapy may translate to better clinical outcomes: for HCC, achieving complete necrosis of a tumor could prevent progression or bridge a patient to liver transplantation; for metastatic CRC, substantial tumor shrinkage might convert an unresectable tumor into a resectable one, enabling potentially curative surgery.

#### Melanoma: proof of concept

Intratumoral/intralesional immunotherapy has seen some early successes in melanoma. The first FDA-approved intratumoral agent was T-VEC, a GM-CSF–expressing oncolytic herpesvirus. In a phase III trial for advanced melanoma, intratumoral T-VEC significantly improved durable response rates (16% vs 2% with control) and overall response rate (26% vs 6%) compared to subcutaneous GM-CSF (9). Another intralesional agent, PV-10 (rose bengal disodium), also showed promising activity in melanoma. A phase II study of PV-10 injections in refractory^1^ metastatic melanoma reported an objective response in 51% of injected lesions, with bystander tumor regression in some non-injected lesions and the occurrence of locoregional blistering. The complete response rate was 26% (10). Intratumoral administration of approved systemic therapies has also proved efficacious. A phase I study of intratumoral ipilimumab and IL-2 in patients with advanced melanoma found injected lesions demonstrated a local response in 67% of patients and an abscopal effect in 89%. Overall response rate was 40% and clinical benefit rate 50%, with most patients exhibiting enhanced systemic immune response associated with clinical outcomes (11).

Combining intratumoral immunotherapy with systemic immune checkpoint inhibitors (ICI) can synergistically enhance anti-tumor immunity. In advanced melanoma, intratumoral T-VEC paired with CTLA-4 blockade (ipilimumab) nearly doubled the objective response rate compared to ipilimumab alone (39% vs 18% objective response rate [ORR]) without added toxicity (12).

#### Uveal melanoma

Uveal melanoma (UM) demonstrates high predilection for hepatic spread, with liver metastases observed in nearly 90% of patients. Locoregional therapies show potential for eliciting a treatment response. For example, a review found liver-directed therapy demonstrated a significant survival advantage over systemic pharmacotherapy in UM, with a median overall survival of 14.6 months versus 9.3 months and a median progression-free survival of 5.2 months versus 2.8 months (13). Limited, yet promising data also exists for thermal ablation treatment of UM hepatic metastases using radiofrequency ablation (RFA), laser-induced thermotherapy (LITT), hepatic intra-arterial chemotherapy (HACT), transarterial chemoembolization (TACE) and selective internal radiation therapy (SIRT) (14). Overall, melanoma (both cutaneous and uveal) has provided evidence that local tumor injections can induce tumor regressions and immune activation in patients.

#### Other cancers

Outside of melanoma, other solid tumors have proven more resistant to intratumoral therapy. Melanoma demonstrate higher response rates to ICIs and are commonly referred to as “hot tumors”, with higher level of immune infiltrates and/or an Interferon (IFN) signature indicative of a T-cell-inflamed phenotype. Solid tumors (i.e., prostate and pancreatic cancers) typically show lower immune infiltrates and are often referred to as “cold tumors”, making effective treatment more challenging (15).

Multiple trials have investigated direct intratumoral injections into primary solid cancers outside of the liver. For example, early trials of intratumoral injections in pancreatic cancer have demonstrated safety but only sporadic efficacy (16). A phase II trial of intratumoral administration of ONYX-015, a replication-selective adenovirus, in patients with refractory head and neck cancer found only modest evidence of antitumoral activity. (17) A phase 1 trial of neoadjuvant intratumoral cisplatin for Stage IV Non-Small Cell Lung Cancer (NSCLC) was halted by the authors for unknown reasons (18).

Despite setbacks, there have been pockets of early success. A Phase I clinical trial of intratumoral injection of the oncolytic virus, HF10 for unresectable locally advanced pancreatic cancer found three partial responses and four stable diseases out of nine subjects (19). A phase I study evaluated intratumoral delivery of a recombinant nonpathogenic polio–rhinovirus chimera in patients with recurrent supratentorial grade IV malignant glioma. The authors found overall survival reached 21% in 24 months, compared to an average of 4% for patients with this same brain tumor undergoing standard treatment (20). Likewise, a phase 1 nonrandomized trial evaluated intratumoral injection of mRNA-2752 and pembrolizumab for high-risk ductal carcinoma, finding 8 of 10 patients responded to treatment and 3 patients had complete responses (21).

Oncology studies are moving more toward synergistic effects, as combining systemic and loco-regional therapies may elicit anti-tumor responses where monotherapy falls short. Phase 3 data combining trans-arterial chemoembolization (TACE) with systemic immunotherapy and Anti-VEGF/TKI have already shown superiority of such combination strategies in advanced HCC (22). Direct intratumoral injection with or without ablation takes this concept further by offering an even more focal or targeted approach compared to TACE, with known advantages of ablation over TACE both in tumorcidal efficacy and sparing of functioning liver tissue. In another example, STING agonist MK-1454 failed to produce complete or partial responses in clinical trials for advanced solid tumors when administered intratumorally as monotherapy. However, when combined with immune checkpoint inhibitors, a partial response of 24% was observed, with reductions in both injected and non-injected lesion sizes (23). A more recent trial showed intratumoral injection had superior systemic immune effects compared to subcutaneous injection (24). Marrying intratumoral agents with immunotherapy or ablation holds potential to trigger durable systemic responses greater than either modality alone. Overall, small sample sizes, early phase trials, inconsistent results, and lack of randomized data make it hard to determine how much intratumoral therapies improve long-term outcomes.

### Unique challenges with liver microenvironment

#### Liver immune tolerance and immunosuppressive milieu

The liver’s unique immune microenvironment is inherently tolerogenic, constantly exposed to food antigens and gut microbes via the portal circulation. Under homeostasis, hepatic antigen-presenting cells (like Kupffer cells and sinusoidal endothelial cells) promote tolerance by releasing IL-10 and expressing checkpoint ligands, which suppress T cell activation (25). In the context of tumors, this baseline immune suppression is greatly amplified. Both primary HCC and liver metastases develop an immunosuppressive tumor microenvironment (TME) rich in regulatory T cells, tumor-associated macrophages (TAMs), and myeloid-derived suppressor cells (MDSCs) that blunt cytotoxic T lymphocyte activity (26, 27). The net effect is a “cold” TME wherein anti-tumor immune responses are muted and tumors evade surveillance. Clinically, this is reflected in the poor response rates to immunotherapy observed in liver cancers. For example, patients with liver metastases often have inferior outcomes on checkpoint inhibitors compared to those without liver involvement, owing to the liver’s role as an “immune-privileged” organ (26). Indeed, recent studies have shown that liver metastases actively co-opt hepatic tolerance mechanisms – in murine models, metastatic tumors in the liver can “siphon” activated CD8^+^ T cells from circulation and eliminate them via FasL-expressing macrophages, leading to systemic T cell depletion and immunotherapy resistance (28). This evidence underscores that any intratumoral therapy in the liver must contend with a highly suppressive immune milieu that readily dampens T cell responses.

#### Prevalence of MDSCs and immune suppression in liver tumors

Myeloid-derived suppressor cells (MDSCs) are highly enriched in liver tumors and are major drivers of immune tolerance in both HCC and liver metastases (29). By releasing arginase, NO, IL-10, and other suppressive mediators, they inhibit T-cell and NK-cell activity and facilitate tumor immune evasion (30–32). High intratumoral MDSC levels correlate with poor survival and reduced responsiveness to anti–PD-L1 therapy in HCC (33). Other immunoregulatory cells, including TAMs and Tregs, add to this suppression through TGF-β, IL-10, and PD-L1 expression (34, 35). Liver stromal cells, including hepatic stellate cells, further amplify suppression by producing IL-6, IL-10 and IDO, promoting Treg expansion and converting monocytes into MDSCs (25). This establishes a reinforcing loop of immunosuppression that limits the efficacy of intratumoral therapies.

Given the dominance of MDSCs and other suppressive elements in liver tumors, intratumoral therapies often require combination approaches, such as pairing with checkpoint inhibitors or MDSC/Treg-modulating agents, to achieve meaningful immune activation (26, 36).

#### Physical barriers: high intratumoral pressure and aberrant vasculature

Liver tumor physical characteristics strongly limit intratumoral therapy distribution. Hepatic tumors, particularly HCC, commonly exhibit elevated interstitial fluid pressure (IFP) driven by rapid proliferation, abnormal angiogenesis, and poor lymphatic drainage (37). In HCC, cirrhosis and portal hypertension further raise tumor pressure, compressing microvessels (37). This vascular collapse impairs perfusion, restricting drug penetration and immune-cell trafficking (38). As a result, therapeutics administered intratumorally can often remain localized near the injection site or track along low-resistance paths, rather than distributing. Irregular and dysfunctional vasculature in HCC and liver metastases compounds these barriers, leaving portions of the tumor poorly perfused or isolated (38). Consequently, large or multifocal lesions can be difficult to treat with single-site injections.

To address these challenges, studies have evaluated pressure-enhanced or catheter-based infusion systems to overcome IFP and improve agent penetration (39), as well as image-guided multi-site injections and drug-eluting depots to achieve more homogeneous intratumoral coverage (40). Overall, the high IFP and abnormal vasculature of liver tumors mean that effective intratumoral therapy often requires engineering delivery approaches that modify pressure gradients or use sustained-release carriers, as simple needle injection is typically insufficient (38).

#### Gut–liver axis crosstalk and intratumoral immunotherapy

The gut and liver maintain a bidirectional immunological dialogue that can profoundly influence the outcomes of localized (intratumoral) cancer therapies. Microbial translocation from a leaky gut – the passage of bacteria or their products into portal circulation – exposes the liver’s immune cells to microbial antigens and endotoxins, which in turn shapes a tolerogenic hepatic milieu (41).Indeed, because the liver is continually bathed in gut-derived antigens via the portal vein, it has evolved dominant immune tolerance mechanisms (e.g. IL-10-secreting Kupffer cells and regulatory T cell induction) to prevent overactivation in response to commensal microbes (26). In the context of intratumoral immunotherapy, this gut–liver crosstalk can dampen therapeutic efficacy: intestinal barrier disruption and dysbiosis drive hepatic immune tolerance processes that suppress systemic anti-tumor immunity (42, 43). For example, enriched Gram-negative pathobionts can chronically trigger TLR4 signaling in the liver, recruiting immunosuppressive myeloid cells and promoting IL-10 production, which creates a liver environment that counteracts the inflammatory T cell responses induced by local tumor therapies (41). While a healthy commensal microbiota helps calibrate immune tone, dysbiosis and microbial translocation skew this balance toward systemic immunosuppression that undermines intratumoral therapeutic efficacy (42).

#### Immunosuppressive cell infiltration in tumors

The accumulation of regulatory immune cells – notably regulatory T cells (Tregs), MDSCs, and M2-polarized TAMs – in the tumor microenvironment creates a suppressive niche that can blunt immune responses induced by intratumoral therapies (44). These cells express high levels of immune checkpoint molecules and secrete anti-inflammatory factors to locally paralyze effector lymphocytes. For instance, TAMs release suppressive cytokines such as interleukin-10 (IL-10) and transforming growth factor-β (TGF-β) and display checkpoint ligands like PD-L1 on their surface, a combination that directly inhibits the cytotoxic activity of tumor-infiltrating T cells (45). Similarly, MDSCs curtail T cell function through multiple mechanisms: they upregulate PD-L1 to engage T-cell PD-1 and induce T-cell anergy (46), produce IL-10 and TGF-β (which promote Treg development and dampen antigen presentation), and deplete nutrients or release reactive species (via arginase-1 and iNOS), all of which contribute to suppression of cytotoxic T lymphocyte activity (47). These myeloid cells can additionally recruit and expand Tregs within the tumor using CCR5-binding chemokines and cytokines, further reinforcing the local immune tolerance (46). The net result of this infiltrate is an immunosuppressive network that expressly blunts intratumoral therapy-induced anti-tumor immunity. By expressing checkpoints, secreting suppressive cytokines, and inactivating effector T cells, Tregs, MDSCs and TAMs collectively dampen the cytotoxic T-cell response that intratumoral immunotherapies seek to elicit (45, 46).

### Liver microenvironment and its impact on intratumoral therapies

#### Liver microenvironment impact on oncolytic viruses

Oncolytic virotherapy is a promising intratumoral approach for liver tumors, but its efficacy is strongly shaped by the liver’s immunosuppressive microenvironment. Oncolytic viruses (OVs) selectively lyse tumor cells and stimulate local immunity, and intratumoral delivery is favored in HCC to bypass rapid hepatic viral clearance and achieve high local titers. However, the tolerogenic liver TME—characterized by TGF-β, IL-10, and checkpoint signaling—can blunt OV-induced immune activation, limiting durability of response (27, 48).

To counter this, ‘armed’ oncolytic viruses expressing immune modulators, such as TGF-β inhibitors, have been shown to enhance antitumor immunity by overcoming immunosuppressive tumor microenvironments in preclinical models. Intratumoral OV administration also preferentially recruits innate immune cells, such as neutrophils and monocytes, which may accelerate viral clearance or reinforce local suppression before effective T-cell priming occurs (49).

Current strategies therefore emphasize combination regimens, pairing OVs with systemic checkpoint inhibitors to overcome local tolerance and promote systemic (abscopal) responses. Overall, durable efficacy in liver tumors likely requires OVs that actively reprogram the TME, through cytokine expression (e.g., GM-CSF, IL-12, IL-21) or local checkpoint inhibition, rather than relying on tumor lysis alone (27, 50).

#### Liver microenvironment impact on dendritic cell vaccines

Intratumoral dendritic cell (DC) vaccination in liver tumors is constrained by a strongly tolerogenic microenvironment. Hepatic DCs typically exhibit an immature or suppressive phenotype, characterized by IL-10 secretion, IDO expression, and promotion of Treg expansion, resulting in poor antigen presentation (25). In liver tumors, DCs are often functionally exhausted and retained within the tumor rather than migrating to lymph nodes, limiting effective T-cell priming^2^. As a result, simple intratumoral DC injection is insufficient unless local immunosuppression is actively mitigated.

Clinical strategies have therefore focused on delivering ex vivo–matured, tumor-antigen–loaded DCs combined with transient suppression of dominant inhibitory pathways. The goal is to reduce tumor burden and Treg/MDSC activity, enabling functional DCs to initiate local antigen presentation and enhance tumor-specific T-cell responses, including increased AFP-specific immunity, without added toxicity (51, 52).

Remaining challenges include impaired DC migration from liver tumors to draining lymph nodes and spatial immune exclusion within the tumor core. Approaches under investigation include local delivery of TLR agonists or adjuvants to activate endogenous DCs, and peritumoral rather than intratumoral injection to target immune-rich invasive margins (39). Overall, effective intratumoral DC vaccination in liver tumors requires highly immunogenic DCs, temporary relief of local immunosuppression, and rational combination with locoregional or systemic therapies.

#### Liver microenvironment impact on intratumoral chemotherapy and ablation

Intratumoral chemotherapy and chemoablative therapies in liver tumors are constrained by both physical and immunologic features of the hepatic microenvironment. Elevated intratumoral pressure and dense fibrotic stroma in HCC restrict the spread of injected agents, often leading to incomplete tumor coverage and residual viable tumor at the margins (53, 54). Rapid tumor growth and fibrosis can create high-pressure compartments that injected fluids or thermal energy fail to penetrate, driving interest in pressure-enabled delivery systems to improve intratumoral drug distribution (38). Abnormal tumor vasculature further limits effective drug accumulation, reinforcing the need for locoregional approaches such as chemoembolization.

Although intratumoral chemotherapy or ablation can induce immunogenic cell death, the resulting hypoxia and VEGF release often recruit MDSCs and TAMs as part of a wound-healing response, blunting durable anti-tumor immunity (55). After transarterial chemoembolization (TACE) or thermal ablation, treated HCC lesions commonly become enriched with PD-L1^+^ macrophages and other suppressive myeloid populations, potentially offsetting cytotoxic benefit and enabling tumor regrowth (55, 56).

These limitations have driven combination strategies that pair locoregional therapy with immunomodulation. For multifocal liver disease, intratumoral therapy alone is rarely sufficient, prompting trials combining ablation with cytokines or TLR agonists to shift the post-treatment microenvironment toward immune activation.

### Biomarkers predicting treatment response in liver cancer

Immunotherapy has significantly expanded treatment options for HCC and liver metastases, yet clinical benefit remains highly heterogeneous, underscoring the need for reliable predictive biomarkers for immune checkpoint inhibitors and intratumoral therapies. To date, no definitively validated predictive biomarker exists for HCC (57). Established candidates, including PD-L1 expression, tumor mutational burden (TMB), intratumoral CD8^+^ T-cell density, and circulating cytokines, have shown variable and often inconsistent associations with response, limited by assay heterogeneity, modest effect sizes, and constrained clinical utility (57). These challenges are further compounded by the liver’s intrinsically immunotolerant microenvironment, which can attenuate antitumor immunity and diminish immunotherapy efficacy, particularly in patients with liver metastases, independent of conventional biomarker status (28).

Among tissue-based markers, PD-L1 expression and tumor-infiltrating lymphocytes (TILs) provide biological insight but lack sufficient predictive precision to guide treatment selection in isolation. Clinical trial data demonstrate that responses to immune checkpoint blockade in HCC occur across PD-L1 strata, with benefit observed even in PD-L1–negative tumors (58–60). Similarly, higher baseline CD8^+^ T-cell infiltration and “T-cell–inflamed” gene expression signatures are associated with improved outcomes, whereas immune-excluded phenotypes, often linked to Wnt/β-catenin activation via CTNNB1 mutations, are associated with resistance (61–63). Beyond tissue biomarkers, circulating inflammatory markers such as IL-6, IL-8, composite indices (e.g. CRAFITY score), and dynamic immune ratios (e.g. early changes in neutrophil-to-lymphocyte ratio) have emerged as accessible predictors of outcome, though standardization and prospective validation remain lacking (64–66). Emerging modalities (including ctDNA, circulating tumor cells, gut microbiome profiling, radiomics, and AI-derived imaging biomarkers) offer promising, non-invasive approaches to capture tumor heterogeneity and treatment dynamics, but their clinical adoption is currently limited by technical variability, cost, and insufficient large-scale validation (67–69). Collectively, these data support a shift toward integrated, multimodal biomarker strategies rather than reliance on any single marker, particularly in the context of intratumoral therapies for liver tumors.

### Imaging guidance modalities for intratumoral liver therapies

Image guidance is central to the safe and effective delivery of intratumoral therapies for liver tumors, with ultrasound (US), computed tomography (CT), and magnetic resonance imaging (MRI) each offering distinct advantages and limitations. Ultrasound remains the most widely used first-line modality due to its real-time needle visualization, lack of ionizing radiation, accessibility, and cost-effectiveness, particularly when lesions are clearly visible on sonography (70, 71). Doppler US further enhances procedural safety by identifying peritumoral vasculature and reducing the risk of intravascular injection (72). However, ultrasound performance is limited by operator dependence, reduced visualization of small, deep, or isoechoic tumors, and challenges posed by patient habitus or anatomic constraints (73, 74). When sonographic visualization is inadequate, CT guidance provides high spatial resolution, reliable targeting of lesions in anatomically challenging locations, and the ability to confirm intratumoral drug deposition using contrast-enhanced techniques, albeit with exposure to ionizing radiation and less continuous real-time feedback (75, 76).

MRI offers superior soft tissue contrast and lesion conspicuity, enabling accurate targeting of small or otherwise occult tumors and avoiding radiation exposure, which is particularly advantageous for repeated treatments or select patient populations (77, 78). However, its use is limited by cost, procedural complexity, restricted availability, and specialized infrastructure requirements, confining MRI guidance to select clinical scenarios (79). In contemporary practice, modality selection is increasingly individualized based on tumor size, location, proximity to major vessels, and anticipated treatment frequency. Multimodal strategies, such as pre-procedural MRI or CT planning with real-time US fusion guidance, are commonly employed to overcome modality-specific limitations and improve targeting accuracy (80, 81). Overall, ultrasound serves as the preferred first-line guidance modality for intratumoral liver therapies, with CT and MRI reserved for lesions that are poorly visualized, anatomically high-risk, or require enhanced soft-tissue delineation, underscoring the importance of a flexible, patient- and tumor-specific imaging approach (40, 82).

#### Targeting small or inconspicuous lesions

Imaging modality selection is critical when treating small liver tumors, such as sub-centimeter HCC or tiny metastases. Ultrasound often has limited sensitivity for lesions below 1–2 cm or those lacking distinctive echogenicity (83), while very small lesions on CT may be transiently visible only during specific contrast phases and can be affected by respiratory motion. In contrast, MRI’s superior contrast sensitivity makes it the most reliable modality for detecting and delineating small or otherwise occult lesions, and MRI-based guidance or planning has been shown to improve targeting accuracy for intratumoral therapies when lesions are not visible on US or CT. (73, 84).

In clinical practice, many centers combine modalities by using pre-procedural MRI or CT for lesion localization and image fusion to guide real-time ultrasound–based injection. Fusion imaging overlays the pre-acquired MRI/CT dataset onto live US, enabling accurate targeting of tumors that are not directly visible on sonography and leveraging MRI’s lesion conspicuity with US’s real-time needle guidance (80, 81). Studies have shown that fusion guidance improves targeting of small or deep liver lesions and increases the technical success of ablation or injection procedures (85).

#### Lesions adjacent to large vessels

Liver tumors abutting major vasculature pose heightened imaging and safety challenges, as inadvertent intravascular injection can result in immediate systemic drug dissemination. Ultrasound may have difficulty defining tumor margins adjacent to large blood-filled structures, whereas contrast-enhanced CT and MRI clearly delineate the relationship between the tumor and nearby vessels, enabling precise needle placement relative to the vessel wall (86–88). CT guidance is particularly valuable in this setting, as small test injections of contrast can confirm intratumoral positioning and detect intravascular leakage in real time, allowing prompt needle repositioning if needed (40).

Ultrasound guidance lacks direct visualization of drug distribution but can still be used with careful technique, such as angled needle approaches to direct the injectate away from vessels. When multiple staged injections are required, the absence of ionizing radiation favors ultrasound or MRI over repeated CT guidance. MRI may offer additional benefit when the tumor–vessel interface is subtle, given its superior soft-tissue contrast. In practice, however, most perivascular liver lesions are managed with CT or ultrasound, and studies of locoregional liver interventions have shown that tumors in challenging locations, including those near central vessels, are preferentially treated under CT guidance due to its superior anatomic definition and broader field of view (89).

#### Radiation exposure and repeat treatments

Many intratumoral therapies, including oncolytic viruses and injectable immunotherapies, require multiple treatment sessions over weeks, making cumulative radiation exposure an important consideration when CT guidance is used repeatedly (40). Ultrasound is well suited for serial treatments because it provides real-time guidance without ionizing radiation, allowing repeated procedures without added radiation risk. MRI similarly avoids radiation but is less often used for repetitive sessions due to logistical complexity and limited availability.

When CT guidance is necessary, radiation exposure can be mitigated through low-dose protocols or combined-modality approaches, such as using ultrasound for real-time needle placement with CT reserved for confirmation of critical steps. Studies of liver tumor ablation have shown that such hybrid strategies can maintain procedural efficacy while reducing overall radiation exposure and complication rates compared with CT-only guidance (87). CT serves as a reliable alternative to ultrasound for lesions that are not sonographically visible or are located in high-risk regions. (40, 82).

### Search methodology

Since the FDA approval of TVEC for melanoma, there has not been a new approved intratumoral injection therapy for liver or non-liver indications. This review analyzes intratumoral therapy studies for both primary and metastatic liver tumors conducted over the past 10 years, revealing several trends and insights valuable for shaping future research and clinical adoption strategies. Search criteria included randomized, controlled clinical trials across any phase, occurring over the past 10 years, evaluating intratumoral therapies for either primary (HCC) or metastatic liver cancer (as a monotherapy or combination therapy), with published methods as well as treatment efficacy details. In the following sections we summarize key trials (Phase I–III) since ~2015 that reported efficacy outcomes (tumor response or survival), not just safety, for intratumoral therapy in liver cancer. These representative, major studies either demonstrated the potential for intratumoral therapies (i.e., via outcomes demonstrating efficacy), or the potential limitations with these techniques that require resolution (i.e., trials that did not improve patient outcomes over the standard of care).

## Intratumoral therapies for primary liver cancer

### Intratumoral monotherapy for primary liver cancer

The studies evaluated in this section include oncolytic viruses and cell-based immunotherapies injected percutaneously into HCC lesions, with results ranging from partial tumor responses and disease control to improved survival in select populations. For a summary of studies, see the supplementary appendix.

#### Oncolytic viruses injected into HCC lesions

Pexa-Vec (JX-594, oncolytic vaccinia virus), an engineered vaccinia virus expressing GM-CSF, Pexa-Vec was tested in a randomized Phase II trial as first-line therapy for advanced HCC (90). Thirty patients received intratumoral Pexa-Vec infusions at high or low dose on days 1, 15, and 29. The trial demonstrated objective responses in injected tumors (15% by mRECIST criteria) and non-injected tumors alike, with an intrahepatic disease control rate of 50%. Notably, high-dose Pexa-Vec doubled median overall survival (14.1 vs 6.7 months for low-dose; *P* = 0.02) despite similar tumor response rates (90). This suggested a dose-related efficacy signal. However, a larger Phase III trial (PHOCUS) evaluating sequential Pexa-Vec (intratumoral) followed by sorafenib versus sorafenib alone was negative (91). These results indicated that while intratumoral Pexa-Vec can induce immunologic and tumor responses, it did not ultimately surpass standard systemic therapy in advanced HCC (discussed in more detail in local and systemic combination therapies below).

OBP-301 (Telomelysin, oncolytic adenovirus) is a telomerase-specific oncolytic adenovirus tested by intratumoral injection in advanced HCC. A multicenter Phase I dose‐escalation trial in 20 patients with refractory HCC (conducted in Asia) established an acceptable safety profile up to 6×10^12 viral particles (92). The most common adverse events were transient fever and fatigue. Although no objective tumor responses were observed in this small trial, about half of patients achieved stable disease in the injected lesions (stable disease rate exceeded the objective response rate). Investigators noted histologic necrosis at injection sites and increased CD8^+ T-cell infiltration in post-treatment tumor biopsies, consistent with a localized oncolytic and immunogenic effect. These findings suggest OBP-301 alone had limited tumor shrinkage in late-stage HCC, but it favorably altered the tumor microenvironment, supporting combination strategies in the future (92).

VG161 (multi-armed oncolytic HSV-1 virus) is a next-generation oncolytic herpesvirus encoding four immunomodulators (IL-12, IL-15/IL-15Rα, and a PD-L1 blocker) for potent immune activation (93). A recent multicenter Phase I trial in China enrolled 44 patients with advanced primary liver cancer (40 with HCC) who had failed ≥2 prior systemic therapies (93). VG161 was delivered via imaging-guided intratumoral injections in dose-escalation and expansion cohorts. The therapy was well tolerated with no dose-limiting toxicity; common events were fever and transient cytopenias, with no significant liver function decline. Encouraging efficacy signals were reported: the ORR was 17.7% and disease control rate 64.7% in this heavily pretreated population. Some patients had substantial tumor necrosis, including one conversion from “unresectable” to resectable disease. Notably, even non-injected tumors regressed in some cases, suggesting a systemic abscopal immune effect (when localized therapy, like radiation, causes the regression of tumors in areas of the body not directly treated) (93). VG161 showed a trend of prolonged overall survival in certain subgroups. In particular, patients who had previously received immunotherapy (checkpoint inhibitors) derived significantly longer median survival with VG161 compared to historical outcomes on second-line drugs. This led to VG161 receiving a Breakthrough Therapy designation in China for post–standard therapy HCC^3^. Taken together, intratumoral VG161 demonstrated meaningful anti-tumor activity and immune reprogramming in refractory HCC, positioning it as a promising third-line immunotherapy (94).

#### Intratumoral cell-based immunotherapy

Ilixadencel (allogeneic dendritic cell therapy) consists of pro-inflammatory allogeneic dendritic cells injected into the tumor to stimulate anti-cancer immunity. In 2019, a Phase I trial evaluated intratumoral Ilixadencel in advanced HCC patients (NCT01974661). Seventeen patients were treated: 11 received Ilixadencel monotherapy (at two dose levels of 10 or 20 million cells per injection), and 6 received Ilixadencel alongside systemic sorafenib (95). Injections were given directly into one of the patient’s tumor lesions under imaging guidance. Ilixadencel was well tolerated; the most common side effects were mild fever/chills, with only one treatment-related grade 3 event. Immunological analyses showed 73% of evaluable patients had increased frequency of tumor-specific CD8^+ T-cells in blood post-therapy, indicating activation of anti-tumor immunity. In terms of efficacy, monotherapy Ilixadencel led to one confirmed partial response (in a patient receiving Ilixadencel alone) and stable disease as best response in five additional patients, for an overall disease control in 6 of 15 evaluable cases. The median time to progression was ~5.5 months, with some patients surviving beyond 18–21 months (95). While no dramatic tumor shrinkage occurred in most patients, these outcomes suggested a subset could attain disease stabilization from intratumoral DC therapy.

### Intratumoral + locoregional therapy for primary liver cancer

Over the past decade, several clinical trials have evaluated intratumoral therapies combined with locoregional treatments (as opposed to systemic therapy) in primary liver cancer (primarily HCC). Below we summarize key trials, including their design and efficacy results.

#### Intratumoral chemotherapy with locoregional ablative therapy

Song et al. (96) conducted a controlled trial in 100 HCC patients to test adding intratumoral cisplatin to transarterial chemoembolization (TACE) plus brachytherapy. All patients received TACE and CT-guided implantation of ^125^I radioactive seeds (brachytherapy); the experimental arm additionally received intratumoral multipoint injections of cisplatin during the procedure (96). This combination significantly improved tumor control and survival outcomes vs the control arm (TACE combined with ^125^I seed implantation therapy without intratumoral injection). The tumor volume reduction averaged 38.6% in the cisplatin-injection group versus 27.4% in control, and alpha-fetoprotein levels fell more sharply with cisplatin (a surrogate for tumor burden). At one year, only 2 patients in the cisplatin group had died of metastases, compared to 8 in the control group (P < 0.05). No significant added toxicity was observed, and the authors concluded that TACE plus brachytherapy was effective for HCC, with even greater efficacy when intratumoral cisplatin was included (96).

#### Intratumoral dendritic cell vaccines with radiotherapy

Another innovative strategy is using intratumorally delivered immunotherapy to boost the effects of local tumor destruction. Kitahara et al. (97) reported a randomized phase II trial in Japan testing intratumoral autologous dendritic cell (DC) injections after radiofrequency ablation (RFA) (97). Thirty patients with small HCC (solitary or ≤3 nodules, <3 cm) received curative RFA and were then randomized to either standard DC therapy or DCs activated with OK432 (a streptococcal immunostimulant) before injection. Notably, all patients received intratumoral DC vaccination, so the comparison assessed enhanced DC activation. The trial demonstrated a clear efficacy signal: the OK432-stimulated DC group had a significantly longer median recurrence-free survival (RFS) of 24.8 months versus 13.0 months in the control DC group (P = 0.003). This translates to an RFS prolongation of ~11.8 months with the immunostimulatory DC injection. Although overall survival did not differ (medians ~68–73 months, P = 0.78), the recurrence delay is clinically meaningful. There were no severe toxicities from the intratumoral immunotherapy; only mild, transient fevers occurred in some patients. This study suggests that post-ablation intratumoral DC vaccines can induce anti-tumor immune effects that delay HCC recurrence (97).

These human results corroborate earlier preclinical and phase I findings. In a 2011 pilot, the same group delivered OK432-activated DCs during transarterial embolization for HCC and observed prolonged recurrence-free survival (97, 98). The RFA trial confirms the approach in a curative-setting: by injecting DCs directly into the ablated tumor site (an antigen-rich environment), an *in-situ* vaccine effect is achieved, enhancing T-cell responses against HCC antigens (97). The success of this strategy has paved the way for combining local tumor ablation with various intratumoral immunotherapies in HCC.

#### Intratumoral immunotherapy with external beam radiation

Building on the abscopal (immune-mediated) effects of radiotherapy, investigators have combined external beam radiotherapy (EBRT) with intratumoral immunotherapeutics. A recent phase I/II trial at Mayo Clinic evaluated intratumoral autologous DC injection after high-dose conformal EBRT in unresectable liver tumors (HCC and iCCA). In the phase I portion, 8 patients (4 HCC, 4 iCCA) received percutaneously injected DCs into the tumor after EBRT (99). The treatment was well tolerated, with only mild post-injection symptoms (e.g. nausea, injection-site pain). Importantly, tumor responses were observed: the objective response rate was 50%, with 4 of 8 patients achieving a partial response by imaging. Patients who achieved stable disease or PR had a median progression-free survival of 11.6 months (range 4–49 months), which is encouraging in this advanced setting. These preliminary results indicate that IT DC therapy + EBRT can induce meaningful tumor regression in a subset of patients. Correlative immune analyses suggested that patients with longer PFS had distinct immune gene expression signatures, hinting that immune mechanisms underline durable disease control. Based on phase I success, the trial’s phase II is ongoing, now adding systemic checkpoint inhibitors (e.g. atezolizumab) on top of EBRT + DC to further boost efficacy (99). Even prior to adding systemic therapy, the combination of locoregional radiation and intratumoral DC vaccine showed promising anti-tumor activity in the liver.

### Intratumoral + systemic therapy for primary liver cancer

In the last decade, several clinical trials have explored intratumoral therapies combined with systemic treatments in HCC.

#### Oncolytic viral therapy plus systemic treatment

PHOCUS was a global phase III trial testing pexastimogene devacirepvec (Pexa-Vec), an oncolytic vaccinia virus injected intratumorally, combined with systemic sorafenib versus sorafenib alone in advanced HCC. Participants were advanced, unresectable HCC patients with no prior systemic therapy. Pexa-Vec was given as intratumoral injections into liver tumors followed by sorafenib, while the control arm received sorafenib alone (91). The primary endpoint was overall survival (OS). The trial enrolled 459 patients (234 combo vs 225 control). At interim analysis in 2019, median OS was 12.7 months in the Pexa-Vec + sorafenib arm vs 14.0 months with sorafenib alone, with no improvement in time to progression or response rates. The combination failed to show any OS benefit; in fact, outcomes trended worse in the combo arm, and the trial was halted early for futility. ORR was ~19% vs 21% (combo vs control) and disease control rate (DCR) ~50% vs 57%, indicating no efficacy advantage. Serious adverse events were more frequent with the virus + sorafenib (54% vs 36% in control), with liver failure being a common severe toxicity. The conclusion was that intratumoral Pexa-Vec combined with sorafenib did not improve survival or response over standard therapy in advanced HCC, underscoring that oncolytic virus monotherapy was insufficient in this setting and that newer approaches (e.g. adding immune checkpoint inhibitors) might be needed (91).

Speculated reasons for this failure included that intratumoral Pexa-Vec was deployed in a heavily immunosuppressive, advanced HCC population and paired with sorafenib, a multikinase inhibitor with known immunosuppressive and hepatotoxic effects, potentially blunting virus-induced immune priming (91). In addition, limited intratumoral dosing coverage in multifocal disease and the absence of immune checkpoint blockade likely prevented conversion of local viral oncolysis into a durable, systemic antitumor immune response (100).

#### Oncolytic adenovirus (H101) + nivolumab – pilot study

A Chinese single-arm pilot study by Yi et al. (101) evaluated an oncolytic adenovirus (H101) delivered by intratumoral injection in combination with systemic nivolumab (anti–PD-1) in patients with refractory advanced HCC. These patients had advanced, unresectable HCC that was resistant to prior therapies. The study was open-label (phase I) with 18 patients treated; H101 injections were given into accessible tumor lesions and nivolumab infusions every 2 weeks, for up to 12 months or until progression (101). Among 18 evaluable patients, the ORR was 11.1% (2 of 18 patients achieved a partial response) and disease control rate 38.9% (including 5 stable disease). While the response rate was modest, the median overall survival reached 15.0 months and 6-month survival rate was 88.9% in this heavily pre-treated population. Median progression-free survival was short (2.7 months), indicating most patients progressed, but a subset derived prolonged benefit (two PR patients survived >30 months). Treatment was generally well tolerated: the most common adverse effect was mild fever from the virus (100% of patients) and injection-site pain; importantly, no grade 3–4 toxicities were observed. Investigators noted that some responses were seen in lesions outside the injected tumor (suggesting an abscopal effect), and intratumoral virus therapy might help overcome PD-1 inhibitor resistance in select patients. This pilot demonstrated feasibility and a signal of efficacy for intratumoral H101 virotherapy + anti–PD-1 in refractory HCC, with an ORR ~11% and good tolerability (101). Larger studies would be needed to confirm any survival benefit.

#### *In situ* immunotherapy (immunomodulators) plus systemic ICI

Liang et al. (102) reported a phase I *in situ* vaccination approach using poly-ICLC (a Toll-like receptor 3 agonist, aka Hiltonol) injected directly into an HCC tumor (and intramuscularly for systemic immune priming) combined with systemic nivolumab in unresectable HCC. This was a proof-of-concept, single-arm trial in Taiwan with *N* = 4 patients, each receiving intratumoral (and intramuscular) poly-ICLC on a schedule alongside nivolumab infusions (102). Although primarily a safety study, efficacy was evaluated by tumor response and alpha-fetoprotein (AFP) tumor marker levels. The combo was safe and well-tolerated with no dose-limiting toxicities in all four patients. Notably, 2 of 4 patients (50%) achieved objective responses: 1 complete response (CR) and 1 partial response (PR) by mRECIST criteria. The patient with CR showed disappearance of viable tumor, including eradication of a cancerous portal vein thrombus, and one PR patient exhibited an abscopal effect. Both responders had marked declines in serum AFP levels correlating with tumor regression. The other two patients had progressive disease. While the sample is very small, these results suggest that intratumoral TLR3 agonist can potentiate checkpoint immunotherapy, yielding deep and systemic responses in some HCC patients. Intratumoral poly-ICLC + nivolumab showed encouraging efficacy (50% response rate) in this tiny cohort (102). This *in situ* immunotherapy approach appeared effective in a subset of unresectable HCC, justifying further investigation in larger trials.

## Intratumoral therapies for metastases to the liver

### Intratumoral monotherapy for metastases to the liver

Over the past decade, several clinical trials have evaluated intratumoral therapies as monotherapies for advanced liver malignancies (unresectable primary liver tumors or cancers metastatic to the liver).

#### Oncolytic HSV-1 (T-VEC) – liver metastases (and advanced HCC)

Hecht et al. reported two Phase Ib/II trials injecting T-VEC into liver tumors of patients with unresectable HCC or various solid tumors metastatic to the liver (103, and 104). In the larger and more recent trial, monotherapy cohorts (T-VEC alone), no objective responses (0% ORR) were observed (NCT02509507). The virus was generally tolerable via image-guided intrahepatic injection, but there were procedure-related complications (e.g. bleeding after biopsy/injection) in a few cases (104). By contrast, combination with pembrolizumab showed low response rates (~8–14%), and overall the trial concluded that intratumoral T-VEC (with or without PD-1 blockade) had limited efficacy in this liver-directed setting (104).

#### Oncolytic HSV-2 (OH2) – GI metastases in liver

Zhang et al. (105) carried out a multicenter Phase I/II trial of OH2, a genetically engineered HSV-2 oncolytic virus (expressing GM-CSF), injected into tumors of patients with advanced solid cancers (including colorectal and esophageal cancers with liver metastases) (105). Among 40 patients treated with OH2 monotherapy, 5% (2 patients) achieved partial responses (in metastatic colorectal and esophageal cancer), with those responses lasting over 11–14 months. No dose-limiting toxicities occurred with monotherapy, and treatment was well tolerated aside from transient mild fevers (105). The study concluded that single-agent OH2 was well-tolerated and showed signs of durable antitumor activity in selected patients (prompting further development, including combination trials).

#### Clostridium novyi-NT oncolytic bacteria – various solid organ tumors (including liver)

Janku et al. (106) conducted a first-in-human Phase I trial of intratumoral Clostridium novyi-NT spore injection. C. novyi-NT is an obligate anaerobic bacterium that germinates in hypoxic tumor cores, causing tumor cell lysis. Twenty-four patients with treatment-refractory solid tumors (some with liver tumors) received a single intratumoral spore injection in a dose-escalation design (106). Tumor lysis of the injected mass was radiographically observed in 42% of patients across doses. At the recommended dose (1×10^6 spores), 41% of evaluable patients had a measurable tumor size reduction, and 86% achieved at least stable disease considering both injected and non-injected lesions. Notably, the therapy can provoke significant inflammatory effects; dose-limiting toxicities included severe *Clostridial*-related events (grade 4 sepsis in 2 patients and gas gangrene in 1 patient with very large tumors) (106). Nevertheless, these results show antitumor activity of C. novyi-NT in humans, with some tumor regressions, supporting further studies (including combination approaches).

While oncolytic viruses and other novel agents can induce local tumor responses – and in some cases systemic immune effects – results have been mixed. Safety profiles also vary. Viral therapies were generally well tolerated (105), but the bacterial therapy carried higher risks at effective doses (106). Ongoing research is exploring modifications and combinations (e.g. adding immunotherapy) to improve efficacy while maintaining safety.

### Intratumoral + systemic therapy for metastases to liver

Recent clinical trials have explored combining intratumoral therapies with systemic treatments in patients with metastatic liver tumors (regardless of the primary cancer origin). Below, we summarize key interventional trials (Phase I–III from ~2015–2025) that reported efficacy outcomes (not just safety).

#### TLR9 agonist (vidutolimod) + checkpoint inhibitors in MSS colorectal liver metastases

Margalit et al. conducted a Phase I trial in microsatellite-stable metastatic colorectal cancer (MSS mCRC) with liver metastases, combining intratumoral injections of the TLR9 agonist vidutolimod with systemic dual checkpoint blockade (nivolumab + ipilimumab) and radiotherapy. Nineteen patients received a priming dose of subcutaneous vidutolimod, three intratumoral vidutolimod injections into hepatic metastases, and stereotactic radiosurgery, alongside nivolumab/ipilimumab (107). The combination was poorly efficacious: no objective responses were observed (0% response rate) aside from one patient with high tumor mutational burden. Immune analyses showed some cytokine changes (e.g. increased CXCL10), but there was no meaningful clinical efficacy, and significant liver toxicity was noted in certain cohorts (107). This highlighted the challenge of overcoming immunotherapy resistance in MSS mCRC liver metastases.

#### Oncolytic HSV-1 Virus (T-VEC) + pembrolizumab in liver metastases/HCC

An open-label Phase Ib/II trial (NCT02509507) investigated T-VEC (oncolytic HSV-1 virus) injected directly into liver tumors, combined with systemic pembrolizumab (anti–PD-1) (104). The study enrolled patients with unresectable HCC or solid tumors with liver metastases. Reviewed in the prior section covering studies evaluating Intratumoral monotherapy for metastatic liver cancer, this trial had both an intratumoral monotherapy cohort as well as an intratumoral + systemic therapy cohort, providing several treatment modalities for efficacy analysis. In dose-escalation, some patients received T-VEC alone and others T-VEC plus pembrolizumab; an expansion phase treated various tumor types with the combination (104). Efficacy was limited: T-VEC monotherapy yielded 0% ORR, and T-VEC+pembrolizumab achieved ORRs of only ~8% in non-HCC tumors and 13.6% in HCC patients. In the expansion (53 patients across tumor types), ORRs ranged 0–20% depending on tumor type. The combination did not significantly improve outcomes over historical controls, and the trial concluded that intrahepatic T-VEC with pembrolizumab did not provide substantial benefit in this population. Notably, some procedure-related hepatic hemorrhages occurred due to biopsy/injection, underscoring risks of intratumoral injections in the liver (104).

#### Rose bengal intratumoral ablation (PV-10) with concurrent immunotherapy in uveal melanoma liver metastases

PV-10 (rose bengal disodium) is an injectable ablative immunotherapy that can be delivered intralesionally. In an ongoing Phase I study (NCT00986661), patients with metastatic uveal melanoma confined to the liver received intratumoral PV-10 injections into liver metastases; many also received concurrent systemic immunotherapy (checkpoint inhibitors) per standard of care (108). Updated data in 2023 showed a 32% ORR in injected liver lesions (25 evaluable patients), including 4% complete responses. Disease control rate was 64%, and the treatment was well tolerated with no ≥Grade 3 toxicity attributable to PV-10^4^. These hepatic responses in a traditionally immunotherapy-resistant cancer (uveal melanoma) are promising. A subset of patients receiving PV-10 alongside immune checkpoint blockade appeared to have prolonged survival (median OS ~30.6 months in M1a uveal melanoma; M1a disease treated with PV-10 and concurrent nivolumab/ipilimumab experienced median OS of 50.0 months). On the strength of this signal, an expansion cohort is planned to formally combine intratumoral PV-10 with ipilimumab + nivolumab to assess potential synergy. While preliminary, this trial suggests that intratumoral chemoablation can induce meaningful responses in liver metastases and might augment systemic therapy in an immunogenic way.

## Analysis of efficacy differences among intratumoral therapies for liver tumors

### Oncolytic viruses: design complexity and immune activation

Oncolytic viruses have shown variable efficacy in liver tumors, largely influenced by their genetic design and payload. Oncolytic viruses selectively replicate in tumor cells and cause direct lysis while igniting local anti-tumor immunity, even within the liver’s highly immunosuppressive microenvironment. Viral infection of HCC cells triggers immunogenic cell death, releasing tumor antigens and danger signals (PAMPs/DAMPs) that recruit dendritic cells and other immune effectors despite the presence of suppressive MDSCs and regulatory T cells (27). Oncolytic virus infection can reprogram the tolerogenic liver tumor milieu by inducing a pro-inflammatory shift, polarizing macrophages to an M1 phenotype and reducing the numbers or activity of MDSCs and Tregs, thereby lifting these inhibitory brakes on anti-tumor T cells (100). Furthermore, many oncolytic viruses are armed with immunostimulatory genes (e.g. GM-CSF) to bolster local immune cell infiltration and activation, helping to overcome the immunosuppressive effects of MDSCs and Tregs in HCC (109).

Early-generation viruses like T-VEC were engineered to express a single immunomodulator (granulocyte-macrophage colony-stimulating factor, GM-CSF) (110). In contrast, newer constructs carry multiple immune-activating genes to amplify anti-tumor responses. For example, the oncolytic herpesvirus VG161 encodes IL-12, IL-15, IL-15Rα, and a PD-1/PD-L1 blocking fusion protein (94), a multi-faceted design intended to enhance local and systemic immunity. These enhancements can significantly reshape the tumor microenvironment – VG161’s *in situ* expression of cytokines led to increased intratumoral CD8^+^ T and NK cells and improved tumor control in a phase I HCC trial (94). By comparison, simpler oncolytic viruses may provoke a more modest immune response; T-VEC’s monogenic design yielded a ~16% durable response rate in melanoma (111), and the vaccinia-based virus Pexa-Vec (JX-594, carrying GM-CSF) failed to significantly prolong survival in a phase III HCC study (91). These outcomes highlight that drug design differences, such as encoding multiple immunomodulators versus one, may be a determinant of efficacy for intratumoral virotherapy in the liver (93, 110). More complex viruses like VG161 aim to overcome HCC’s immunosuppressive milieu and have shown the ability to rescue tumors that were resistant to prior systemic treatments (94), whereas earlier-generation viruses often fell short in such refractory cases.

### Intratumoral cell-based immunotherapies: targeting tumor antigens and microenvironment

Cell-based therapies injected directly into liver tumors (or via locoregional infusion) have demonstrated potent anti-tumor effects, but their efficacy can differ due to targeting and microenvironment factors. Chimeric antigen receptor (CAR) T-cells directed at liver tumor antigens like glypican-3 (GPC3) can induce significant tumor regressions in HCC, provided the target is abundantly expressed (112). Early-phase trials of GPC3 CAR-T in advanced HCC reported manageable safety and some durable responses, but also highlighted challenges – for instance, systemic CAR-T infusions led to cytokine release syndrome (CRS) in most patients (including a fatal case) when tumor burden and T-cell expansion were high (113). This prompted innovations in design and delivery: next-generation CAR-T cells are being “armored” with additional genes to resist the immunosuppressive tumor microenvironment (e.g. a dominant-negative TGF-β receptor or secretion of IL-7 and CCL19 to attract T cells and dendritic cells) (113, 114). Such modifications have improved intratumoral T-cell expansion and tumor infiltration. Delivering cell therapies intratumorally or via hepatic artery has the added benefit of concentrating effector cells in the tumor and limiting systemic exposure. This can enhance efficacy and safety compared to intravenous infusions, which may disperse cells and trigger off-tumor effects. The need for antigen presence and the variability of each patient’s tumor immune landscape mean outcomes can be mixed, but ongoing trials with armored CAR-T and NK cells are showing improved response rates in heavily pre-treated HCC patients by directly counteracting the local suppressive factors (113).

### *In-situ* immunomodulators: local immune stimulation and combination strategies

*In-situ* immunotherapy involves injecting immune-stimulatory agents directly into the liver tumor to ignite a localized anti-cancer immune response. Examples include cytokine therapies, pattern-recognition receptor agonists, and other immunomodulatory compounds delivered intratumorally. These agents can differ in efficacy based on how powerfully they activate the immune system and how they are used in combination with other treatments. For instance, intratumoral delivery of IL-12 has shown strong anti-tumor effects in liver tumor models – IL-12 gene therapy or mRNA injected into HCC can drive a potent TH1 immune response (high IFN-γ and cytotoxic T-cell activity) with only mild systemic toxicity (115). In preclinical studies, combining local IL-12 with other therapies (e.g. radiation) led to dramatic regression of large orthotopic HCC tumors, illustrating the potential of this approach (116). Likewise, injecting Toll-like receptor 9 (TLR9) agonists into liver tumors can convert an immunosuppressive microenvironment into an immunopermissive one by activating dendritic cells and macrophages to produce IL-12, type I interferons and other cytokines (117). While TLR9 agonist monotherapy has shown limited efficacy in patients (likely because the immune stimulation alone is not enough to overcome robust tumor defense), greater efficacy is observed when such *in-situ* immunomodulators are combined with systemic treatments. For example, an early trial of intratumoral TLR9 agonist with systemic checkpoint inhibitors demonstrated cooperative anti-tumor activity, as the local CpG injection primed the tumor for T-cell attack that the checkpoint blockade then amplified (117) Overall, therapies like intratumoral IL-12, TLR agonists, or STING agonists can induce local tumor regressions and systemic immunity (abscopal effect), but their success in liver tumors often hinges on multi-modal strategies. Differences in injection regimens (single-agent vs combination, one-time vs repeated dosing) heavily influence outcomes. Trials in solid tumors (including liver cancers) suggest that repeated intratumoral doses or combining local immunotherapy with PD-1/PD-L1 inhibitors are needed to achieve durable responses, whereas single injections of an immunostimulant may yield only transient or partial effects (110, 117). Thus, the efficacy of *in-situ* immunomodulators is closely tied to how they are integrated into broader treatment plans and how effectively they activate immune cells within the tumor microenvironment.

### Patient factors influencing efficacy

In addition to the therapy design, real-world efficacy differences arise from who is treated and how the treatment is delivered. Intratumoral therapies for liver tumors are often trialed in patients with advanced, refractory disease who have exhausted standard treatments (118). Such patients frequently have tumors that evolved mechanisms of immune evasion (e.g. high levels of regulatory T-cells and myeloid-derived suppressor cells in HCC) (41). This baseline immune resistance can blunt the effectiveness of a given intratumoral therapy. Indeed, efficacy may vary with prior immunotherapy exposure and response. For example, a recent oncolytic virus trial in HCC noted that individuals who had previously been sensitive to checkpoint inhibitor therapy showed enhanced tumor regression with intratumoral virus injections, compared to those who never responded to immunotherapy (94). This suggests baseline patient immune status (such as tumor-infiltrating lymphocyte levels or prior immune priming) may play a role in the efficacy of subsequent treatments. Aligning the treatment parameters with the patient’s condition (immune status, tumor burden) may be as important as the drug design itself for maximizing the efficacy of intratumoral therapies in liver cancers (110, 119).

### Dosing/administration factors influencing efficacy

Differences in how intratumoral treatments are given also contribute to efficacy variability. Some therapies require intensive dosing schedules to maintain immune pressure on the tumor. T-VEC, for instance, has been administered as an initial priming dose followed by biweekly intratumoral injections, a strategy that achieved continuous local inflammation and tumor shrinkage in melanoma (8, 120). In liver tumor trials, however, not all therapies may have been delivered optimally. For example, the Pexa-Vec phase III HCC study employed a limited number of virus injections before transitioning to sorafenib, which may have curtailed its potential efficacy (91). Higher or more frequent doses could improve tumor responses but are often limited by safety concerns (especially in HCC patients with cirrhosis who have less hepatic reserve). Moreover, precise delivery techniques (e.g. ultrasound-guided injections into multiple tumor sites) and combining intratumoral therapy with systemic agents may improve outcomes. Current trials are exploring combination regimens (for example, repeated intratumoral cytokine injections paired with checkpoint blockade) to synergistically enhance efficacy.

## iCCA vs HCC: microenvironmental differences and therapeutic implications

HCC and intrahepatic cholangiocarcinoma (iCCA) are regarded as two distinct forms of liver cancer, reflecting fundamental differences in their underlying pathogenesis, histopathological characteristics, clinical prognosis, and responses to adjuvant therapies. Comparative context with HCC is illuminating, as the two primary liver cancers differ in biology and in their responses to intratumoral therapies. Biologically, iCCA arises from biliary epithelium and is characterized by a pronounced desmoplastic stroma with abundant cancer-associated fibroblasts and matrix, whereas HCC originates from hepatocytes in the context of chronic liver disease (121, 122). These distinct milieus shape the immune landscape of the tumors. Single-cell analyses have confirmed that T cells in HCC are generally more suppressed and exhausted than T cells in iCCA tumors (123). Chronic inflammation (e.g. viral hepatitis or NASH in cirrhotic livers) can drive T-cell exhaustion in HCC, whereas iCCA’s dense stromal barrier tends to exclude immune cells outright or skew them toward immunosuppressive phenotypes (124). Notably, myeloid cell populations also diverge: for example, a unique EGR1^+^ macrophage subset is enriched in HCC but not in iCCA, reflecting different tumor–myeloid interactions (123).

These microenvironmental differences have practical ramifications for intratumoral therapies. In HCC, longstanding experience with locoregional treatments (including intratumoral ethanol ablation and transarterial chemoembolization) has shown that well-vascularized HCC nodules can be controlled locally, improving survival in early-stage disease (90, 125). By contrast, iCCA is less often amenable to such local control – it typically presents as a solitary large mass or multifocal disease at diagnosis, with worse baseline prognosis than HCC (5-year survival <20%) (126). Traditional locoregional therapies like embolization have had limited impact on iCCA outcomes, partly due to lower tumoral blood supply and the physical drug penetration barrier posed by the tumor stroma. Cholangiocarcinoma presents a highly complex tumor microenvironment consisting of malignant cells, abundant inflammatory infiltrates, and cancer-associated fibroblasts embedded within a dense desmoplastic matrix. Treatment is primarily surgical resection, if possible, followed by chemotherapy (127, 128). This underlines why innovative intratumoral approaches are so needed in cholangiocarcinoma.

Early evidence suggests that when these therapies can be effectively delivered, iCCA tumors can mount immune-mediated regressions. For example, one small phase 1 study (n=7) treated cholangiocarcinoma with an oncolytic virus (chimeric poxvirus CF33-hNIS, as a single agent or in combination with pembrolizumab), resulting in an objective response rate of 14% and a disease control rate of 86%. One patient with cholangiocarcinoma in this study experienced an immunological complete response. Another small phase 1 study of both HCC and iCCA (HCC, n=4; iCCA, n=4) treated patients using radiation-primed intratumoral DC injections (high-dose conformal EBRT followed by intratumoral injections of autologous DCs). Overall response rate was 50% (4 partial responders). Among patients who had stable disease and partial response, median progression free survival was encouraging at over 11 months (99).

Despite positive preliminary results, the frequency and predictability of such outcomes in iCCA remain lower than in HCC, where immunotherapy and locoregional treatments have a more established record of extending survival. Moving forward, a nuanced understanding of each tumor type’s microenvironment will be crucial. Tailoring intratumoral therapies to iCCA’s biology, for instance, by combining therapies to penetrate the desmoplastic barrier and reverse immunosuppression, may help achieve the kind of success that has been seen in HCC. Recognizing the immunosuppressive tumor microenvironment of iCCA, emerging strategies pair intratumoral therapies with complementary treatments to enhance antitumor immunity. The rationale is that intratumoral therapy can inflame the tumor and increase antigen presentation, making otherwise “cold” iCCA lesions more responsive to systemic immunotherapy. Continued comparative studies of iCCA vs HCC are likely to yield insights into optimizing intratumoral treatment strategies for both malignancies (123).

## Next steps: research priorities for intratumoral therapy in the liver

### Expanding combination strategies

Emerging evidence presented in this paper suggests that rational combination regimens will be pivotal in maximizing the impact of intratumoral therapies for liver cancers. In both HCC and liver metastases, intratumoral immunotherapy is being explored as an adjunct to systemic treatments, with the goal of augmenting anti-tumor immunity without added systemic toxicity. Promising strategies pair intratumoral agents with immune checkpoint inhibitors or other immunotherapies. For example, delivering an oncolytic virus into liver tumors alongside anti–PD-1 therapy has yielded tumor responses even in previously immune-resistant HCC, hinting that local therapy can potentially reverse checkpoint inhibitor resistance (101). Combination approaches are not limited to systemic immunotherapy; integrating intratumoral immunotherapy with locoregional treatments (e.g. thermal ablation or embolization) is another priority. For example, a phase 1/2 study of tremelimumab in combination with ablation in patients with advanced HCC found ~26% achieved a confirmed partial response, with six-week tumor biopsies showing increase in CD8+ T cells for patients demonstrating a clinical benefit. Duration of responses was noteworthy, lasting 7 to 19 months for positive responders (129). Such findings support the concept of using local therapy to create an *in-situ* vaccine effect, converting the treated tumor into a nidus of systemic anti-tumor immunity. Notably, this approach may be especially valuable for liver metastases, which often foster an immunosuppressive microenvironment that blunts systemic immunotherapy responses (25). By locally re-activating immune surveillance in hepatic lesions, intratumoral therapies could help overcome the liver’s tolerogenic niche and improve the efficacy of checkpoint inhibitors in patients with metastatic disease. Collectively, these early signals underscore the need for larger, well-controlled clinical studies to validate the durability, reproducibility, and generalizability of these combination strategies, and to define optimal sequencing, patient selection, and biomarker correlates necessary for their safe and effective integration into routine clinical practice.

### Developing standardized administration procedures

An equally critical research priority is the establishment of standardized, reproducible administration procedures for intratumoral therapies in the liver, as technical variability remains a major and often underappreciated determinant of therapeutic efficacy and safety. Technical consistency is critical, as variations in injection method can lead to uneven drug distribution or off-target effects (130). Best practices must be defined for key parameters such as injection volume relative to tumor size, number of injection sites for larger tumors, and frequency of dosing. The use of image guidance (ultrasound or CT) is strongly recommended during percutaneous intratumoral injections to ensure accurate needle placement and adequate perfusion of the agent throughout the tumor. Recent consensus guidelines from the Society for Immunotherapy of Cancer (SITC) include illustrated instructions for proper intratumoral immunotherapy delivery in various organs (including liver lesions) (131). These emphasize meticulous documentation of each injection, recording the exact site, depth, and technique, to facilitate reproducibility across institutions. Going forward, establishing such uniform protocols will be crucial to fairly evaluate intratumoral agents in clinical trials and to optimize outcomes in practice, given the liver’s unique challenges (vascularity, risk of bleeding, and potential for drug leakage into circulation).

### Identifying robust biomarkers

As mentioned previously, both HCC and liver metastases currently lack reliable predictive biomarkers of response to immunotherapy. Traditional markers that work in other cancers (for example, PD-L1 expression or high tumor mutational burden) have shown limited predictive utility in HCC (132), likely due to the distinct biology of liver tumors. This has spurred efforts to discover new biomarkers reflecting the immunological and molecular features of the treated tumor. Candidate biomarkers under evaluation are diverse and include tumor-infiltrating T cells, circulating cytokines and immune markers, gene mutation status and expression signature changes, gut microbiome elements, circulating tumor DNA levels, as well as radiologic and imaging biomarkers. Observations hint that dynamic immune biomarkers – e.g. a falling neutrophil-to-lymphocyte ratio – could signal effective tumor immune activation in the liver (64). Genomic features are another avenue: for instance, specific tumor gene expression profiles (such as an “inflamed” interferon-γ signature) or oncogenic pathways (e.g. β-catenin activation status) might inform likelihood of response or resistance to intratumoral immunotherapy (133). Likewise, imaging-based biomarkers merit exploration. Advanced functional imaging or radiomic analysis of tumor lesions could noninvasively detect immunologic changes post-therapy (for example, increased PET uptake in activated immune cells or radiographic evidence of necrosis and edema corresponding to immune-mediated tumor damage) (134–136). Moving forward, clinical trials should integrate systematic tissue biopsies, blood analyses, and imaging studies before and after intratumoral treatment to validate such biomarkers (132). Establishing reliable predictors of response or resistance will enable clinicians to personalize intratumoral therapies, matching the right combination to the right patient, and to monitor early on whether a therapy is hitting its intended immunological targets.

## Conclusions

Despite promising early results, the clinical development of intratumoral therapies remains in its nascent stages. Most published studies are small, single-arm trials with limited long-term follow-up, making it difficult to draw definitive conclusions about durability of response or survival benefit. Variability in injection techniques, drug distribution, and tumor accessibility further complicates standardization and reproducibility. Specific pharmocodynamic data will likely be needed for injected agents to customize appropriate delivery methods. Additionally, while local immune activation is often observed, translating these responses into systemic tumor regression has proven inconsistent. As such, broader validation in randomized studies and clearer patient selection criteria will be essential before intratumoral therapy can be fully integrated into standard care pathways for liver tumors.

A key insight from recent trials is the synergy of intratumoral therapies with other cancer treatments. When integrated into multimodal regimens, intratumoral treatments can amplify therapeutic effects. In melanoma, intralesional oncolytic virus therapy (T-VEC) combined with systemic checkpoint inhibition improved objective response rates compared to immunotherapy alone, with some patients experiencing regression of distant, non-injected lesions (abscopal effect) and no added toxicity (137). Notably, patients who mounted robust tumor-specific T-cell responses after intratumoral vaccinations had higher 5-year relapse-free survival (97). These examples illustrate that intratumoral therapies can synergize with systemic and locoregional modalities to enhance overall anti-tumor immunity (137).

Looking ahead, continued progress in intratumoral therapy will depend on coordinated advances in clinical research, commercial translation, and regulatory strategy. Clinically, priorities include optimizing delivery technologies and combination approaches. Novel image-guided delivery systems and injectable drug depots, such as biocompatible gels that solidify within the tumor, are being developed to enhance targeting accuracy and prolong intratumoral drug retention (40). Refinement of injection schedules and dosing is expected to further improve local distribution and immune activation. Rational combination strategies represent another key focus, including optimal sequencing of intratumoral agents with systemic therapies such as chemotherapy, immune checkpoint inhibitors, or targeted agents. Synergistic integration with locoregional modalities (e.g., TACE, radioembolization, thermal ablation, cryotherapy, pulsed electric field therapies, or focused ultrasound) may further amplify therapeutic efficacy. In parallel, the identification and validation of predictive biomarkers will be essential for patient selection; emerging evidence indicates that patients demonstrating robust intratumoral T-cell activation or favorable immune signatures experience more durable clinical benefit (97). Both molecular and imaging-based biomarkers may ultimately enable a more personalized approach. Finally, careful protocol-level specification of inclusion and exclusion criteria, such as tumor stage, size, and burden, will be critical to reduce heterogeneity and improve interpretability of clinical outcomes, particularly given the inherent variability in immune responsiveness.

Ongoing basic science research into tumor microenvironment modulation and immune priming will continue to inform these developments, but the emphasis remains on translating insights into therapies that tangibly improve patient outcomes. With multidisciplinary progress, intratumoral treatments hold significant promise to enhance the therapeutic arsenal against HCC and liver metastases in the coming years.

## Footnotes

1 The term “refractory” is used in this review according to study-specific eligibility criteria. For example, in early-phase intratumoral studies, refractory disease commonly denotes tumor persistence or recurrence despite prior therapeutic interventions, whereas in advanced hepatocellular carcinoma it typically refers to progression on, or intolerance to, prior approved systemic therapy.

2 The ASCO Post Staff. *Adding dendritic cell vaccination to hepatocellular carcinoma therapy.* The ASCO Post, August 2025. Available at: https://ascopost.com/news/august-2025/adding-dendritic-cell-vaccination-to-hepatocellular-carcinoma-therapy/ (Accessed December 12, 2025).

3 Virogin Biotech. Virogin’s first oncolytic virus VG161 receives breakthrough therapy designation in China. 2024. Available at: https://virogin.com/breaking-news-virogins-first-oncolytic-virus-vg161-receives-breakthrough-therapy-designation-in-china/ (Accessed September 7, 2025).

4 Ryan, C. PV-10 elicits hepatic responses in uveal melanoma with liver metastases. OncLive, September 7, 2023. Available at: https://www.onclive.com/view/pv-10-elicits-hepatic-responses-in-uveal-melanoma-with-liver-metastases (Accessed September 7, 2025).
