# Supplementary material for: Advancements in intratumoral therapies for liver tumors
Source: Front Oncol. 2026 Feb 4;16:1726128. doi: 10.3389/fonc.2026.1726128 (PMC12913102; doi:10.3389/fonc.2026.1726128)
Supplement: Supplementary file 1 [file Table1.docx]

## Supplementary Material

Table 1: Representative Studies Evaluating Intratumoral Monotherapy for Primary Liver Cancer (HCC)

| **Study** | **Patient Population** | **Intratumoral Therapy (Control)** | **Route** | **Study Design** | **Patients (per arm)** | **Duration** | **Subgroup Efficacy** | **Follow-up Duration** | **AE Grading Criteria** | **Primary Outcomes & Efficacy Results** |
| --- | --- | --- | --- | --- | --- | --- | --- | --- | --- | --- |
| Pexa-Vec Phase II – JX-594 oncolytic vaccinia (Heo et al., 2013) | Advanced HCC, unresectable (no prior systemic tx) | Pexa-Vec virus, **High-dose vs Low-dose** (randomized dose-finding) | Intratumoral (image-guided injection into liver tumors) | Open-label **Phase II** (2-arm) – intratumoral Pexa-Vec on Days 1, 15, 29 at two dose levels to assess optimal dose. | N=30 (High n=16, Low n=14) | ~1 month treatment + follow-up (median OS ~year) | No specific subgroup analysis included | Disease control at 8 weeks; median follow-up not explicitly reported; 1 year, 18-month survival estimates reflect longitudinal observation | National Cancer Institute Common Terminology Criteria for Adverse Events of the adverse events | **Tumor response:** 15% ORR by mRECIST; 50% disease control in injected lesions. Responses seen in non-injected tumors as well, confirming systemic effect. **Survival:** High-dose prolonged median OS to 14.1 mo vs 6.7 mo in low-dose (*HR* 0.39, *P*=0.02). **Significance:** Established proof-of-concept for oncolytic virotherapy in HCC (dose-dependent efficacy). |
| OBP-301 Phase I –“Telomelysin” oncolytic adenovirus (Heo et al., 2023) | Advanced HCC, sorafenib-refractory (Asia-Pacific) | OBP-301 virus (telomerase-selective Ad; **no control**) | Intratumoral (US-guided tumor injection; repeat dosing) | Open-label **Phase I** dose escalation – 5 cohorts of escalating virus dose; multiple IT injections to a liver tumor in each patient. | N=20 (single-arm; dose cohorts of ~3–6 each) | ~3 months on trial (eval at 6 weeks, follow-up) | Median times to progression (TTP) = 19.10 (cohort 1), 4.10 (cohort 2), 8.10 (cohort 3), 4.30 (cohort 4), and 8.10 weeks (cohort 5).  Median OS = 40.86 weeks (cohort 2), 12.86 weeks (cohort 4), 23.00 weeks (cohort 5). | Up to 12 weeks after the last injection | Common Terminology Criteria for Adverse Events (CTCAE) version 4.0 | **Safety:** Well tolerated up to 6×10^12 vp (MTD not exceeded); common AEs fever, fatigue, transient cytopenias. **Efficacy:** No confirmed partial responses; however **stable disease** in injected tumors in >50% of patients (vs 0% ORR). Histology of post-treatment tumors showed virus-induced necrosis at injection sites and increased CD8+ T-cell infiltration. **Significance:** Monotherapy yielded disease stabilization but limited tumor shrinkage; demonstrated localized tumor killing and immune activation, informing future combination strategies. |
| VG161 Phase I – Multi-armed HSV-1 oncolytic virus (IL-12/IL-15/PD-L1 block) (Li et al., 2025; Shen et al, 2025) | Advanced HCC, **refractory** (failed ≥2 lines, incl. immunotherapy) | VG161 virus monotherapy (**no control** in Phase I; BTD based on China data) | Intratumoral (CT-guided injection; multiple doses) | Multicenter **Phase I** (China) – 5 dose-escalation cohorts + expansion; intratumoral VG161 injections (up to 3 doses) in patients with no options. (Breakthrough status granted after separate Phase II showing OS benefit vs control.) | N=44 (Phase I; 40 HCC in efficacy analysis) | ~2–3 months treatment; follow-up ongoing (long-term OS tracked) | VG161-treated pts had significantly longer OS than controls (median OS: 9.40 months versus 4.70 months; P = 0.03)  Pre-checkpoint inhibitors (CPI) > 3 months vs ≤ 3 months  - Median PFS: 3.6 months vs 1.8 months (P = 0.04)  -Median OS: 17.3 months vs 7.4 months (P = 0.04**)**  Patients who underwent post-systemic therapy (PST) following VG161 treatment had enhanced survival (median OS:  20.10 months versus 8.80 months for those who received no further  anti-cancer treatment; P = 0.03) | Median follow-up 7.0 months (range: 1.7–26.3  Months) | National Cancer Institute Common Terminology Criteria for Adverse  Events v5.0 | **Safety:** No dose-limiting toxicities; transient low blood counts; no significant liver toxicity. **Efficacy:** **ORR 17.7%**, **DCR 64.7%** in heavily pretreated HCC. Notable tumor necrosis observed; 1 patient’s tumor downsized to operable. Uninjected tumors also regressed (implying systemic immune effects). Patients previously exposed to checkpoint inhibitors showed improved outcomes; VG161 *significantly prolonged OS* in immunotherapy-experienced patients compared to historical controls. **Significance:** First-in-human data show VG161 can rescue a subset of refractory HCC with meaningful responses and immune remodeling, supporting it as a promising third-line immunotherapy. |
| Ilixadencel Phase I – Allogeneic dendritic cell vaccine (Rizell et al., 2019). | Advanced HCC, BCLC B/C (mixed prior therapy; some on sorafenib) | Ilixadencel cell therapy (DCs); **no control** arm (monotherapy ± sorafenib) | Intratumoral (US-guided percutaneous injection into one tumor) | Open-label **Phase I** (Sweden) – dose-finding (10×10^6 vs 20×10^6 cells) and combination feasibility. Patients received 2 IT DC injections; 6 patients continued sorafenib concurrently (combination subset). | N=17 (Monotherapy n=11; Combo n=6) | ~3 months initial evaluation; 12-month survival follow-up | Shorter median OS in patients receiving ilixadencel + sorafenib as first-line therapy (N = 6; 8.6 months) vs ilixadencel monotherapy in second-line setting (N = 7; 10.9 months  Tumor-specific T-cell induction less frequent with combination therapy (50% vs 78%).  Outcomes in first-line monotherapy subgroup heterogeneous, may be influenced by baseline prognostic factors (performance status, AFP level, metastatic burden). | Up to 6 months after last patient's last vaccination; OS ranged from 1.6 to 21.4 months | Common Terminology Criteria, version 4.03. | **Safety:** Favorable tolerability; only one grade 3 event related to treatment (fever/chills most common). **Immune activity:** 73% of patients had increased tumor-specific CD8 T-cells post-therapy. **Efficacy:** 1 patient (9%) achieved **PR** (monotherapy arm), 5 (29%) had **SD** ≥3 mo (overall disease control 40%). Median time to progression was 5.5 months; some patients survived >1 year. No significant tumor shrinkage in most cases, but prolonged stable disease in several. **Significance:** Provided first evidence that intratumoral DC therapy can activate immunity and delay progression in HCC, laying groundwork for combining intratumoral vaccines with other treatments. |

HCC - hepatocellular carcinoma. ORR - objective response rate. DCR - disease control rate. PR - partial response. SD - stable disease. OS, - overall survival. AEs - adverse events. HSV-1 - herpes simplex virus type 1. BTD - Breakthrough Therapy Designation. DCs -dendritic cells. US – ultrasound. CT -computed tomography, mRECIST - modified version of the RECIST criteria used to assess tumor response. HR - hazard ratio. Ad is - adenovirus. vp - viral particles, IT - intratumoral administration. tx - treatment, mo – months. MTD - maximum tolerated dose. BCLC B/C - stages B and C of the Barcelona Clinic Liver Cancer (BCLC) staging system

Table 2: Representative Studies Evaluating Intratumoral + Locoregional Therapy for Primary Liver Cancer (HCC)

| **Study** | **Patient Population** | **Therapies (Intratumoral + Locoregional)** | **Route** | **Study Design** | **Patients (per group)** | **Study Duration** | **Subgroup Efficacy** | **Follow-up Duration** | **AE Grading Criteria** | **Primary Outcomes & Results** |
| --- | --- | --- | --- | --- | --- | --- | --- | --- | --- | --- |
| TACE + Cisplatin Injection (Song, et al, 2021) | Unresectable HCC; both hypovascular and hypervascular tumors (China) | *Intratumoral:* Cisplatin (multi-point intralesional injections) *Locoregional:* TACE + ^125^I brachytherapy seeds (control: TACE + ^125^I only) | Percutaneous CT-guided tumor injections; intra-arterial chemoembolization; intratumoral seed implant | controlled trial comparing TACE+brachy vs TACE+brachy+Cisplatin IT | N = 100 total (50 control, 50 combo therapy) | ~1 year follow-up (outcome assessed at 12 months) | No specific subgroup analysis included | ~1 year follow-up | AE grading system not specified | **Tumor response:** Greater tumor shrinkage with IT cisplatin (38.6% vs 27.4% volume reduction). **AFP tumor marker:** Larger decline in combo arm (significantly lower AFP than control). **Survival:** 1-year survival improved – deaths due to metastasis 4× lower in IT cisplatin group (2 vs 8 deaths, *P*<0.05). **Conclusion:** Both groups effective, but adding intratumoral cisplatin yielded significantly better tumor control and survival. |
| RFA + DC Vaccine (Kitahara *et al.*, 2020) | Early-stage HCC (solitary or ≤3 tumors <3 cm); mostly HCV-related; post-ablation setting (Japan) | *Intratumoral:* Autologous dendritic cells (DC) vaccine injected into ablated tumor bed (experimental DCs pre-stimulated with OK432 vs control DCs) *Locoregional:* Curative radiofrequency ablation (RFA) of tumor lesion | Ultrasound-guided intratumoral needle injection (post-RFA) | Randomized phase II trial of RFA in all patients, with 1:1 randomization to DC vs OK432-stimulated DC | N = 30 (14 standard DC, 16 OK432-DC) | Median follow-up ~5 years (patients followed ≥2 yrs) | Patients with significantly increased tumor-associated antigen–specific T-cell responses had markedly higher 5-year RFS rates (50.0% vs. 7.7%; P = .030) | Follow-up to assess long-term recurrence and survival outcomes, Kaplan–Meier analysis indicating median RFS outcomes at approximately 2 years, 5-year RFS comparisons among responders vs. non-responders | National Cancer Institute Common Toxicity Criteria | **Recurrence-Free Survival:** Significantly prolonged with OK432-activated DCs: median RFS 24.8 vs 13.0 months (**P = 0.003**). 5-year RFS rate ~50% (OK432-DC) vs 7.7% (control). **Overall Survival:** ~68–73 months median; no significant difference (n.s.). **Immune Response:** Enhanced tumor-specific T-cell responses observed after DC injections, correlating with longer RFS. **Safety:** Well-tolerated; no grade 3–4 toxicity, only mild fever in ~40%. **Conclusion:** Feasible and safe. Intratumoral DC therapy after RFA **significantly delayed HCC recurrence** vs control. |
| Phase I/II – EBRT + Intratumoral DC (Wu et al, 2024) | Unresectable primary liver tumors (HCC (n=4) or iCCA (n=4) in phase I); Child-Pugh A/B, no extrahepatic spread (USA) | *Intratumoral:* Autologous dendritic cell vaccine (in situ matured, injected per tumor) *Locoregional:* High-dose conformal external beam radiotherapy (EBRT) to all tumor sites | Ultrasound-guided intratumoral injection (post-EBRT, repeated each treatment cycle) | Phase I/II single-arm trial; Phase I evaluated safety & efficacy of EBRT + IT DC; Phase II expanding with systemic immunotherapy | Phase I: N = 8 (each patient as their own control vs historical data); Phase II ongoing | Median PFS follow-up ~12 months in Phase I (range up to 49 mo); results reported after all 8 completed ≥1 cycle | Exploratory subgrouping by progression-free survival showed median PFS 6.45 months in PFS-S (<12 months) vs 20.2 months in PFS-L (≥12 months); among SD/PR patients median PFS was 11.6 months (range 4–49). | Follow-up duration not explicitly stated; PFS outcomes reported with observed PFS range of 4–49 months in SD/PR patients | AE grading criteria not specified | **Tumor Response:** 50% objective response rate (4/8 patients achieved partial responses). Remaining had stable disease; no progressions during initial eval. **Progression-Free Survival:** Among patients with SD/PR, median PFS = 11.6 months (range 4–49). Some patients sustained disease control >1–2 years. **Safety:** Well tolerated; no dose-limiting toxicities. Common AEs were transient nausea or pain post-injection. **Immune findings:** Treated tumors showed T-cell clonal expansion; one Phase II patient (with added immunotherapy) even achieved complete response. **Conclusion:** **Feasible with promising efficacy** – EBRT + intratumoral DC induced notable response rate and prolonged disease control in unresectable HCC. |

HCC - hepatocellular carcinoma, TACE - transarterial chemoembolization, CT - computed tomography, AFP - alpha-fetoprotein, RFA - radiofrequency ablation, DC - dendritic cells, OK432- bacterial immunostimulant used to activate dendritic cells. EBRT - external beam radiotherapy. iCCA - intrahepatic cholangiocarcinoma, PFS - progression-free survival, PR - partial response, SD - stable disease, AEs - adverse events

Table 3: Representative Studies Evaluating Intratumoral + Systemic Therapy for Primary Liver Cancer (HCC)

| **Study** | **Patient Population** | **Intratumoral + Systemic Therapy (Control)** | **Route of Administration** | **Study Design** | **Patients (per arm)** | **Study Duration** | **Subgroup efficacy** | **Follow-up Duration** | **AE Grading Criteria** | **Primary Outcomes & Results** |
| --- | --- | --- | --- | --- | --- | --- | --- | --- | --- | --- |
| PHOCUS Pexa-Vec (Phase III) (Abou-Alfa et al., 2024) | Advanced unresectable HCC, no prior systemic therapy | Pexa-Vec oncolytic vaccinia virus + sorafenib vs sorafenib alone | Intratumoral virus injections; sorafenib orally (systemic) | Randomized open-label Phase III trial (intratumoral virus given weeks before starting sorafenib in combo arm) | N=459 (234 combo, 225 control) | ~3.5 years (trial stopped early at interim analysis in Aug 2019 for futility) | Patients with more advanced disease (i.e., BCLC Stage C and baseline tumor size ≥75th percentile) experienced longer OS when treated with sorafenib alone, compared to pexa-vec plus sorafenib combination.  Subgroup analyses suggested correlation between number of doses administered and OS.  Patients on the pexa-vec plus sorafenib arm who received full 3 doses of pexa-vec experienced median OS of 16.3 months (95% CI: 13.04, 20.96), compared to 1.8 months (95% CI: 1.31, 12.68) for patients who received one dose of pexa-vec | Safety follow-up visit at least 28 days after last study treatment. Patients contacted ~ every 4 weeks. Median follow-up 8.0 months in pexa-vec plus sorafenib arm,8.3 months in sorafenib arm | AE grading criteria not specified | **Primary endpoint:** Overall Survival. **Outcome:** *No improvement* in OS with combo (median 12.7 vs 14.0 months for sorafenib alone, p=n.s.). ORR 19.2% vs 20.9%, no significant difference; trial terminated early due to lack of efficacy. Combo arm had higher toxicity (e.g. 54% serious AEs vs 36%). |
| H101+ nivolumab  (Yi et al., 2024). | Refractory advanced HCC (failed prior therapy, incl. ICIs) | H101 oncolytic adenovirus + nivolumab (single-arm study, no separate control) | Intratumoral virus injections; nivolumab IV infusion | Open-label single-arm pilot study (Phase I) at Fudan Univ. Cancer Center (China) evaluating feasibility and efficacy | N=18 treated (of 21 screened) | ~2 years enrollment (Mar 2020–Mar 2022); follow-up median ~15 mo OS | Extended OS (>2.5 years) observed in responders with low AFP levels. | Median follow-up was 11.89 months (range 1.94-32.13 months) | National Cancer Institute Common Terminology Criteria (version 5.0) | **Primary endpoint:** Objective Response Rate. **Outcome:** ORR 11.1% (2/18 PRs); Disease control rate 38.9%. **Median OS** 15.04 months; 6-mo survival 88.9%. **Median PFS** 2.7 months. No grade 3–4 toxicity (main side effect: mild fever). Promising signals of efficacy in a subset (prolonged OS in responders), suggesting the virus may help overcome PD-1 resistance. |
| Poly-ICLC (TLR3 agonist) + nivolumab (Phase I) (Liang et al., 2025) | Unresectable HCC (advanced, not surgical candidates) | Poly-ICLC (TLR3 agonist) + nivolumab (single-arm) | Intratumoral + intramuscular injections of poly-ICLC; nivolumab IV | Phase I single-arm trial (Taiwan) – “in situ vaccine” approach combining local TLR3 immune stimulation with systemic PD-1 blockade | N=4 (all in combination arm) | ~1–2 years (exact duration not stated; results published 2025) | Small sample size; no subgroup evaluation | Followed for response and safety for up to 2 years or until withdrawal, death, or study termination | Common Terminology Criteria for Adverse Events (CTCAE) version 5.0 | **Primary endpoint:** Safety (Phase I); **Secondary:** tumor response. **Outcome:** Safe with no DLTs. **Efficacy:** 50% ORR in 4 patients – *1 CR and 1 PR* achieved. CR patient had complete tumor and portal vein thrombus regression; PR patient showed an abscopal tumor shrinkage effect. Both responders had large AFP declines, indicating robust tumor kill. These results demonstrate potent immune-mediated effects in two patients, albeit in a very small sample. |

HCC - hepatocellular carcinoma. IV - intravenous administration, PR- partial response, CR- complete response, ORR -overall response rate. PFS - progression-free survival, OS - overall survival. n.s. - not significant, AEs -adverse events, DLTs - dose-limiting toxicities, AFP - alpha-fetoprotein. ICIs - immune checkpoint inhibitors.

Table 4: Representative Studies Evaluating Intratumoral Monotherapy for Metastatic Liver Cancer

| **Study** | **Patient Population** | **Therapy (Control)** | **Route** | **Study Design** | **Patients (Groups)** | **Study Duration** | **Subgroup efficacy** | **Follow-up Duration** | **AE Grading Criteria** | **Primary Outcomes & Efficacy Results** |
| --- | --- | --- | --- | --- | --- | --- | --- | --- | --- | --- |
| T-VEC in Liver Tumors (Phase Ib/II in HCC & mets (NCT02509507) (Hecht et al., 2025) | 74 patients in 2 cohorts: **Group A:** various solid tumors with liver metastases; **Group B:** unresectable **HCC**  Advanced solid tumors with liver metastases; included cohort of unresectable HCC. | Talimogene laherparepvec (T-VEC, oncolytic HSV-1) – tested alone (dose escalation) and with pembrolizumab | Intratumoral (intrahepatic injections into liver lesions, via imaging guidance) | Phase Ib (**dose-escalation**) + Phase II (**expansion**) basket trial. Part 1: T-VEC monotherapy (multiple doses) ± pembrolizumab; Part 2: combination expansion in select tumors. | N=74 (Part 1: T-VEC mono *n=28*, T-VEC+pembro *n=46*; Part 2: combo *n=53*) | Enrollment ~2016–2020; treatment until progression (injections q2–3 weeks up to 5 doses in Part 1) | ORR (95% CI) 0% (0.0, 30.8)-20.0% (0.5, 71.6) across 5 tumor types, with 16.7% (95% CI: 3.6, 41.4) for triple-negative  breast cancer with largest sample size (n = 18**)** | Therapy every 12 weeks (±28 days) following safety follow-up visit until death, patient withdrawal, or up to ~ 24 months after date of last patient enrolled | AE grading criteria not specified | **Primary focus:** Safety/DLTs – *3 patients with DLTs* (2 on T-VEC alone – grade 3 liver enzyme elevation/ pain; 1 on combo – hepatitis). **Efficacy:** *Monotherapy T-VEC:* ORR 0% (no responses). *T-VEC+Pembrolizumab:* ORR 8.3% in non-HCC, 13.6% in HCC. No clear improvement over historical data; **Outcome:** **No significant efficacy** seen with T-VEC alone in liver tumors (trial concluded further development not warranted). |
| OH2 HSV-2 trial – Phase I/II in GI cancers (Zhang et al., 2021). | 54 patients with **metastatic solid tumors** (refractory **colorectal, esophageal**, etc.; all had injectable lesions, including liver metastases) | **OH2 oncolytic HSV-2** encoding GM-CSF – tested as monotherapy and with anti–PD-1 (HX008) | Intratumoral (direct injection into accessible tumors; ultrasound guidance for liver lesions) | Multi-center Phase I (**dose escalation**) and Phase II (**dose expansion**) trial in China. Parallel cohorts: OH2 alone vs OH2 + anti–PD-1 antibody. | N=54 (OH2 alone *n=40*, OH2+HX008 *n=14*) | Trial duration ~Apr 2019 – Sep 2020 (Phase I/II). Patients treated until PD or intolerance; responders followed for durability | Durable objective responses in patients with metastatic esophageal cancer and rectal cancer noted | Median follow-up for efficacy evaluation 5.41 months (range: 0.46–19.48+ months) | NCI Common Terminology Criteria for Adverse Events (CTCAE) version 5.0 | **Primary endpoints:** Safety and MTD; **Secondary:** anti-tumor activity (RECIST/iRECIST responses). **Monotherapy Results:** Well tolerated – *no DLTs*. *Efficacy:* 2 of 40 patients (~5%) had confirmed **partial responses** (mismatch-repair-proficient rectal cancer and esophageal cancer metastases) on OH2 alone. These responders had **durable responses** lasting 11.3+ and 14.0+ months. Other patients had stable disease or progression; combo cohort had similar two PRs (but shorter duration). **Outcome:** OH2 **monotherapy induced occasional long-lasting tumor regressions** (PRs) in metastatic GI tumors; **safe profile** (mostly grade 1–2 fevers). |
| C. novyi-NT Bacterial Therapy – Phase I in advanced solid tumors (Janku et al., 2021) | 24 patients with **treatment-refractory tumors** (various types; injectable tumor sites included liver, soft tissue, etc.). Many had large, bulky tumors (median size ~5–8 cm). | **Clostridium novyi-NT** oncolytic anaerobic bacteria (spores) – single intratumoral injection (no control; dose-escalation from 1×10^4 up to 3×10^6 spores) | Intratumoral (CT/ultrasound-guided injection of spores into the tumor core; patients hospitalized for monitoring post-injection) | First-in-human **Phase I dose-escalation** trial (3+3 design) assessing safety (MTD) and preliminary efficacy. Single injection per patient at assigned dose; follow-up imaging and biopsies for response. | N=24 treated (6 dose cohorts; up to 5 patients per dose level) | Patients observed acutely for ~28 days post-injection for DLTs; tumor response assessed at 1–2 months and longitudinal follow-up to evaluate durability. | Antitumor activity observed primarily in patients who developed intratumoral bacterial germination.  Germination + grade ≥3 AEs occurred more frequently in larger tumors | Patients hospitalized for initial monitoring, subsequently followed in clinic twice weekly for 3 weeks, then at 2, 4, 8, and 12 months post-discharge for safety / efficacy assessments | AE grading criteria not specified | **Dose findings:** MTD determined as 1×10^6 spores (higher doses caused dose-limiting toxicity). **Safety:** Notable AEs at high doses – e.g. grade 4 *Clostridial* sepsis (2 patients) and gas gangrene (1 patient) in those with very large tumors. **Efficacy:** *Radiologic tumor lysis* of the injected tumor observed in 10 of 24 patients (42%) across all doses. In 22 evaluable patients, **41%** had a >0% **tumor size reduction** and **86%** achieved **stable disease** or better in overall response (considering injected and non-injected lesions). Some non-injected tumors showed inflammatory changes or shrinkage, suggesting systemic immune effect. **Outcome:** Intratumoral *C. novyi*-NT showed **localized tumor destruction** in nearly half of patients and disease stabilization in most, but with dose-dependent infectious risks. Further trials are exploring refined dosing and combination with immunotherapy. |

T-VEC - talimogene laherparepvec, HCC - hepatocellular carcinoma, HSV-2 - herpes simplex virus type 2, GM-CSF - granulocyte-macrophage colony-stimulating factor, PD-1 - programmed death-1, ORR -objective response rate, RECIST -Response Evaluation Criteria in Solid Tumors, iRECIST - immune RECIST, DLT - dose-limiting toxicity, maximum tolerated dose (MTD). AE adverse event, CTCAE - Common Terminology Criteria for Adverse Events. MTD - maximum tolerated dose, PR - partial response, PD - progressive disease.

Table 5: Representative Studies Evaluating Intratumoral + Systemic Therapy for Metastatic Liver Cancer

| **Study** | **Patient Population** | **Intratumoral + Systemic Therapy (Control)** | **Route** | **Study Design** | **Patients (per arm)** | **Study Duration** | **Subgroup efficacy** | **Follow-up Duration** | **AE Grading Criteria** | **Primary Outcome & Results** |
| --- | --- | --- | --- | --- | --- | --- | --- | --- | --- | --- |
| Intratumoral TLR9 Agonist + Dual Checkpoint – Sheba Medical Center single-institution trial (Margalit et al., 2023). | MSS mCRC with unresectable liver metastases (heavily pre-treated) | Vidutolimod (TLR9 agonist) intratumoral + nivolumab & ipilimumab (no separate control arm; all patients received combo; radiotherapy to tumor included) | Intratumoral injections (liver lesions); s.c. priming dose; IV for nivolumab/ipilimumab; SBRT to lesion | Phase I, open-label, 4 sequential cohorts (varying radiotherapy timing relative to intratumoral therapy). Aim: induce immune activation in liver mets | **N = 19** (single-arm; 4 cohorts of ~4–5 pts each) | ~7-week treatment period + follow-up (3 intratumoral injections over ~6 weeks; cytokines measured at 7±2 weeks) | Only patient who responded subsequently found to have high tumor mutational  burden (TMB) 79 mutations / Mb despite being MMR proficient  and MSS | Patients followed from treatment initiation until disease progression, unacceptable toxicity, or for up to 24 months | National Cancer Institute Common Terminology Criteria for Adverse Events (CTCAE), version 5.0. | **Primary outcome:** Safety and feasibility. **Efficacy:** 0% ORR (no objective responses observed); 1 of 19 patients had a minor response (high TMB case). **Result:** **Not efficacious** – no significant tumor regressions. **High-grade toxicity:** Liver enzyme elevations in later cohorts (Grade 3 hepatitis in up to 75% of pts in one cohort). |
| Oncolytic Virus T-VEC + Pembrolizumab–Phase Ib/II in Liver Tumors – Phase Ib/II in HCC & mets (NCT02509507) (Hecht et al., 2025). | 74 patients in 2 cohorts: Group A: various solid tumors with liver metastases; Group B: unresectable HCC Advanced solid tumors with liver metastases; included cohort of unresectable HCC. | Talimogene laherparepvec (T-VEC, HSV-1 oncolytic virus) intrahepatic injection + pembrolizumab IV (Part 2 combo expansion). *No true control arm* (Part 1 had T-VEC alone vs T-VEC+pembro in non-random cohorts). | Intratumoral (intrahepatic) injection via imaging guidance; IV pembrolizumab. | Phase Ib (dose-finding: T-VEC alone in some pts, then T-VEC+pembro) and Phase II expansion (T-VEC+pembro in 5 tumor-type cohorts). Multi-center study. | **Part1:** 28 (T-VEC monotherapy) + 46 (T-VEC+pembro); **Part2:** 53 (T-VEC+pembro across cohorts). | Conducted ~2016–2021; median follow-up ≈ 12 mo in expansion. Injections given every 2 weeks; pembrolizumab every 3 weeks (per cycle). | ORR (95% CI) 0% (0.0, 30.8)-20.0% (0.5, 71.6) across 5 tumor types, with 16.7% (95% CI: 3.6, 41.4) for triple-negative  breast cancer with largest sample size (n = 18**)** | Therapy every 12 weeks (±28 days) following safety follow-up visit until death, patient withdrawal, or up to ~ 24 months after date of last patient enrolled | AE grading criteria not specified | **Primary outcomes:** Dose-limiting toxicities (Part1) and ORR by mRECIST (Part2). **Results:** *Limited efficacy.* T-VEC alone ORR 0%; T-VEC + pembro ORR ~8.3% in non-HCC, 13.6% in HCC (Part1). In Part2 expansion, ORRs ranged 0–20% (e.g. 16.7% in largest cohort, triple-neg breast). **No significant improvement** over expected pembro-alone rates; combination deemed **not a viable strategy** for HCC or liver metastases. Safety: some injection-related liver hemorrhages (procedure-related); overall AE profile acceptable, similar to known effects of virus and immunotherapy. |
| PV-10 Intralesional Ablation (Phase I) – Uveal Melanoma Liver Mets (Provectus PV-10-LC-01 study) (Ryan, 2023). | Metastatic **uveal melanoma** with dominant liver metastases (M1a disease common). Patients could receive concurrent immune checkpoint therapy. | Intratumoral **PV-10** (rose bengal) percutaneous injection into liver lesions; **Systemic:** allowed standard immunotherapy (44% received anti–PD-1 or ipilimumab+nivolumab concurrently). No separate control arm. | Intralesional (ultrasound-guided liver injection); systemic therapies IV as per standard regimens. | Phase I open-label basket trial (ongoing). Single-arm cohorts for different tumor types; this cohort is uveal melanoma in liver. Endpoints: safety, ORR in injected lesions, survival. | **N = 25** in uveal melanoma liver metastasis cohort. (Phase I expanded after initial enrollments.) | Patients treated in cycles: PV-10 injections could be repeated every ≥28 days; median 2 hepatic lesions injected per patient. Data cutoff for analysis in 2023 (with some patients receiving ≥2 cycles). | 4 patients with M1a experienced durable complete metabolic responses (CMRs); Patients who achieved a CMR accounted for 24% of all patients with M1a disease and 16% of all patients with uveal melanoma. | Follow-up for CMR patients: median 39 months (range, 24.6–61.6) | AE grading criteria not specified | **Outcomes:** **Intratumoral ORR 32%** in injected liver lesions (4% CR + 28% PR); stable disease in additional 32% → **64% disease control**. Some patients had metabolic complete responses on PET. Median overall survival ~30.6 mo in patients with only liver metastases (M1a). **Interpretation:** PV-10 injections induced notable local responses in uveal melanoma liver mets, an otherwise refractory setting. Safety was favorable (no treatment-related Grade ≥3 toxicity). A follow-up trial will combine PV-10 with checkpoint inhibitors (ipi+nivo) to evaluate synergistic efficacy. |

MSS mCRC - microsatellite-stable metastatic colorectal cancer. TLR9 - Toll-like receptor 9, ORR - objective response rate, TMB - tumor mutational burden, SBRT - stereotactic body radiation therapy.,IV - intravenous, s.c. - subcutaneous administration, HCC - hepatocellular carcinoma, HSV-1 - herpes simplex virus type 1, T-VEC -talimogene laherparepvec, Pembro - pembrolizumab, nivo/ipi - nivolumab and ipilimumab, CR / PR - complete and partial response, PET - positron emission tomography, AE - adverse events, mRECIST - modified Response Evaluation Criteria in Solid Tumors, M1- disease refers to metastatic cancer confined to one organ.
